# Interaction of West Nile virus NS5 with orthoflavivirus SLA RNAs and their effects on viral replication and inhibition

**DOI:** 10.1128/jvi.02023-24

**Published:** 2025-08-18

**Authors:** Mandi A. Feinberg, My T. Le, Kassandra L. Carpio, Ekaterina Knyazhanskaya, Alan D. T. Barrett, Kyung H. Choi

**Affiliations:** 1 Department of Biochemistry and Molecular Biology, University of Texas Medical Branch 12338, Galveston, Texas, USA; 2 Sealy Center for Structural Biology, University of Texas Medical Branch 12338, Galveston, Texas, USA; 3 Department of Molecular and Cellular Biochemistry, Indiana University 1772, Bloomington, Indiana, USA; 4 Department of Pathology, University of Texas Medical Branch 12338, Galveston, Texas, USA; University of Kentucky College of Medicine, Lexington, Kentucky, USA

**Keywords:** West Nile virus, orthoflavivirus, NS5 polymerase, RNA promoter, stem-loop A, replication, infection, inhibition

## Abstract

West Nile virus (WNV) is a single-stranded, positive-sense RNA virus in the *Orthoflavivirus* genus, within *Flaviviridae*. The genus encompasses numerous pathogens of public health importance, including WNV, dengue virus (DENV), Zika virus (ZIKV), and Japanese encephalitis virus (JEV). Orthoflavivirus replication depends on the presence of the stem-loop A (SLA) structure in the 5′ untranslated region of the genome. The viral polymerase, NS5, interacts with the SLA and initiates synthesis of the negative-strand RNA. The sequences and secondary structures of SLA and NS5 are highly conserved across orthoflaviviruses, suggesting that the viruses utilize a similar SLA-mediated replication mechanism. Here, we determined the molecular shapes of WNV and JEV SLAs and investigated WNV NS5 interaction with orthoflavivirus SLAs. Although WNV NS5 interacts with DENV, ZIKV, and JEV SLAs in binding assays, only DENV and ZIKV SLAs could replace WNV SLA for viral replication. Next, we found that the top and side loops of SLA are important regions for WNV NS5 interaction. Consequently, when these SLA mutations were introduced into a WNV replicon, genomic replication was greatly reduced. Finally, we tested whether the WNV SLA mimic could inhibit viral replication. The addition of exogenous SLA reduces replication of both WNV replicon and infectious virus, suggesting that exogenous SLA can outcompete the viral SLA for NS5 interaction. Next-generation sequencing data indicate that the presence of exogenous SLA during infection increased the genetic diversity of WNV.

**IMPORTANCE:**

West Nile virus (WNV) causes West Nile disease in humans. Approximately 1 in 150 cases develops serious neurological complications, such as meningitis or encephalitis. Currently, no vaccines or antiviral treatments are available. WNV relies on a conserved RNA element in the genome, known as stem-loop A (SLA), to recruit viral polymerase for replication. We found that WNV polymerase can bind the SLAs of other orthoflaviviruses, including dengue virus (DENV), Zika virus (ZIKV), and Japanese encephalitis virus (JEV). However, only the DENV and ZIKV SLAs supported replication when substituted into a WNV replicon. The failure of the JEV SLA to support WNV replication suggests that efficient replication requires additional virus-specific factors beyond the polymerase-SLA interaction. We then tested whether exogenous SLA could act as an RNA decoy to compete with genomic SLA and inhibit viral replication. The addition of SLA RNA in virus-infected cells significantly reduced viral replication and infection, highlighting the therapeutic potential of viral RNA mimic against WNV.

## INTRODUCTION

West Nile virus (WNV) is a member of the *Orthoflavivirus* genus within the family *Flaviviridae,* which also includes dengue virus (DENV), Zika virus (ZIKV), and Japanese encephalitis virus (JEV), all of which cause human diseases and are considered etiologies of emerging infectious diseases (1). WNV is a mosquito-borne virus that is the causative agent of West Nile fever and West Nile neuroinvasive disease (WNND) in humans (2). The virus is endemic in North America, Central Europe, and the Mediterranean Basin and is the most common mosquito-borne disease in the United States (1, 3). Approximately 1 in 150 individuals infected with WNV develop WNND, leading to meningitis or encephalitis and neurological impairments in survivors (2, 4). While there are at least nine recognized genetic lineages of WNV, only lineages I, II, and III are known to cause clinical disease in humans (1, 5–8). Lineage I is most prevalent around the globe; however, cases of lineage II have been on the rise throughout the Eastern Hemisphere, as with all mosquito-borne diseases (9–11). The first report of lineage III in a human occurred in 2023 in the United States (8). There is currently no vaccine or therapeutic for WNV approved for use in humans (1, 2, 12).

The genome of WNV consists of a single open reading frame flanked by 5′ and 3′ untranslated regions (UTRs), which have a 5′ cap and no poly(A) tail, respectively (3). The positive-sense RNA genome acts as an mRNA and is translated into a polyprotein that is co- and post-translationally cleaved into 10 proteins, 3 structural (capsid C, pre-membrane prM, and envelope E) and 7 non-structural (NS) proteins (NS1, NS2A, NS2B, NS3, NS4A, NS4B, and NS5). Viral replication is carried out by the membrane-associated replication complex consisting of all NS proteins, although enzymatic activities reside in NS3 and NS5 (3, 13, 14). NS3 consists of two domains, the N-terminal protease and C-terminal helicase domains, responsible for polyprotein processing and helicase activities, respectively (3, 13). NS5, the largest and most conserved orthoflavivirus protein, is responsible for replicating the viral genome (1, 3). NS5 is composed of the N-terminal methyltransferase (MTase) domain and the C-terminal RNA-dependent RNA polymerase (RdRp) domain. The MTase domain is responsible for catalyzing the capping of the 5′ end of the viral RNA and methylation of the cap during genomic replication (1, 15, 16). The RdRp domain consists of thumb, fingers, and palm subdomains. The palm subdomain is the most conserved domain among viral RdRps and is the catalytic domain of the polymerase. The fingers subdomain curves to form the template-binding channel and interacts with the thumb subdomain (17). NS5 also supports viral infection by inhibiting the signaling pathway of type I interferon by directly interacting with various components of the pathway (18, 19). Crystal structures of WNV and other orthoflavivirus NS5, either the full-length or individual domains, have been determined (15, 20, 21).

**Fig 1:**
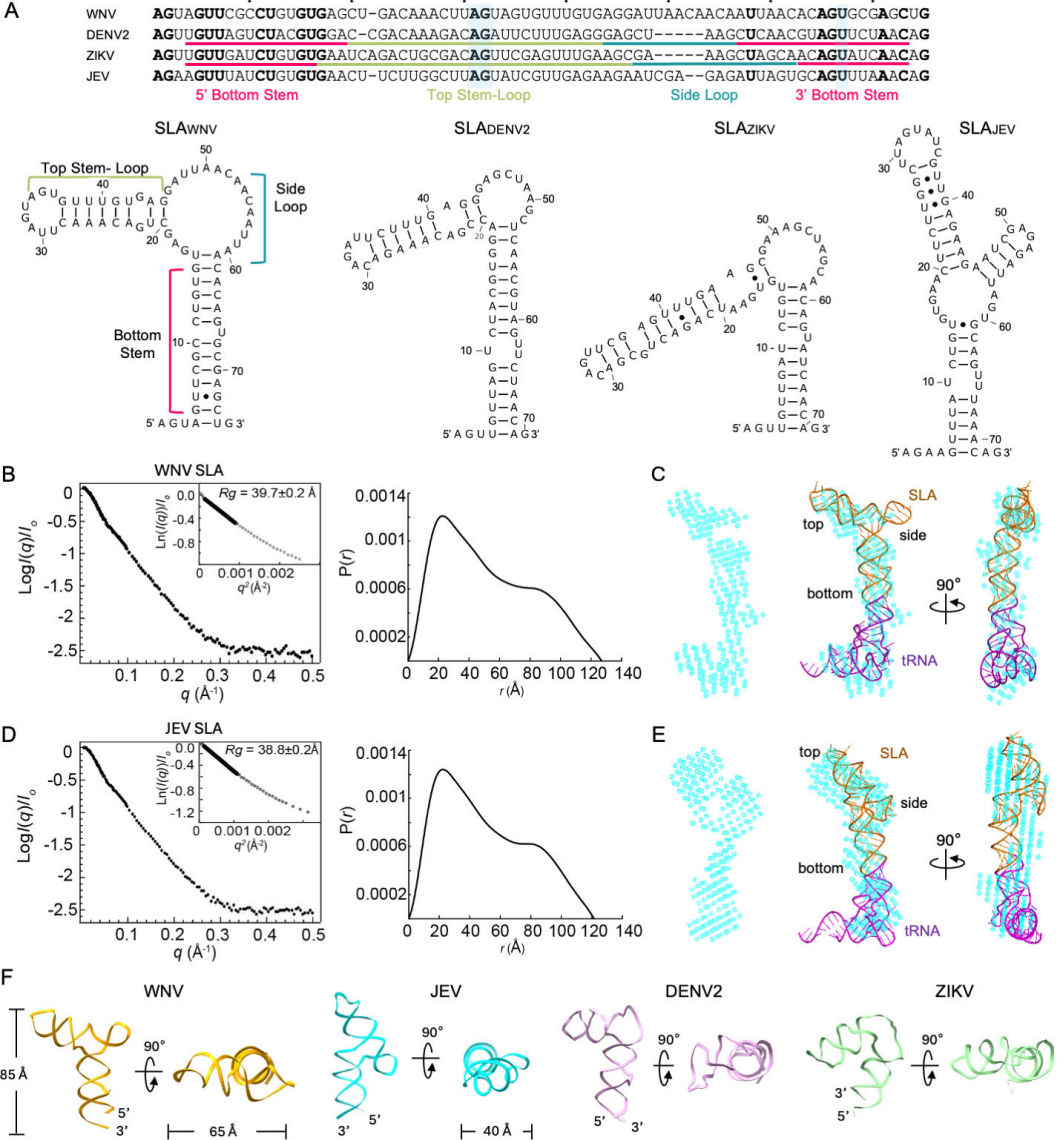
Structure and sequence of orthoflavivirus stem-loop A. (**A**) Sequence and secondary structures of orthoflavivirus SLAs. The SLA sequences from WNV (NCBI accession number, NC_001563), ZIKV (KU527068), DENV2 (NC_001474), and JEV (NC_001437) are aligned. Conserved nucleotides are shown in bold, and the conserved AG motif and U-bulge in orthoflavivirus SLAs are highlighted in blue. The bottom stem, top stem-loop, and side loop are indicated for DENV2 and ZIKV under their sequences (31). Secondary structures of DENV2 and ZIKV SLA were obtained from the crystal structures, and those of WNV and JEV SLA were predicted using the RNAfold web server (32). (**B and D**) Small-angle X-ray scattering analysis of WNV (**B**) and JEV SLA-tRNA (**D**). Scattering profiles of SLA-tRNA collected at 1 mg/mL are shown along with the Guinier plot (left, inset) and the pairwise distribution function, P(r) (right). (**C** and **E**) The overall shapes and SCOPER models for WNV (**C**) and JEV SLA-tRNA (**E**). The molecular shapes generated using the scattering profiles are shown in cyan (left). SCOPER models of SLA-tRNA were overlaid with the envelope (right). SLA is depicted in yellow, and tRNA is in purple. The fit of the scattering data generated from the SCOPER model to the experimental data is shown in Fig. S2C. (**F**) 3D structural models of WNV and JEV SLAs. The small-angle X-ray scattering models of WNV and JEV SLA are shown along with crystal structures of DENV (PDB 7LYF) and ZIKV SLA (PDB 7LYG). The size of WNV and JEV RNAs is indicated.

The 5′ and 3′ UTRs of the WNV genome contain conserved secondary structures that are vital for replication, including the 5′ stem-loop A (SLA), 3′ terminal stem-loop (3′SL), and two sets of complementary sequences at the 5′ and 3′ ends that facilitate the circularization of the genome (5′/3′ upstream AUG region and 5′/3′ conserved sequence) (3, 22–24). SLA formed by the first 73 nucleotides of WNV 5′ UTR functions as a promoter for RNA synthesis by directly binding to the viral polymerase, NS5 (Fig. 1A) (25, 26). Orthoflaviviruses utilize these SLA structures to recognize the viral genome among the host mRNAs within the cytosol (1, 25). Previous studies have shown that removal of the SLA in DENV abolishes viral replication but not viral translation, implicating its role in viral genome synthesis (27). Viral replication is thought to begin with circularization of the viral genome, which inhibits the binding of host ribosomes, thus allowing for the synthesis of the negative-sense RNA strand (22, 28). NS5 then binds to the 5′ SLA and initiates negative-strand RNA synthesis from the 3′ terminus (22, 27, 29, 30). NS5 next uses the negative-sense RNA as a template to synthesize additional positive-sense RNA. The newly synthesized positive-sense RNAs are used to translate additional viral proteins and to be encapsidated in the viral capsids (3, 14, 24).

Orthoflavivirus SLAs share conserved sequence and secondary structure features (25). Recent SLA structures from DENV and ZIKV show that SLA forms a three-way junction structure, consisting of top stem-loop, side loop, and bottom stem (Fig. 1A) (31, 33, 34). The conserved AG motif and U-bulge sequences are located in the top loop and bottom stem. However, the relative orientation of the top stem-loop and bottom stem differs between DENV and ZIKV SLA, suggesting a dynamic nature to their interaction with NS5. Furthermore, RNA protection assays using WNV and DENV SLA–NS5 complexes showed that different SLA nucleotides are involved in NS5 interaction (29, 34). Currently, the structure of WNV SLA is lacking, and thus it is not well understood how WNV SLA interacts with NS5, and whether the sequence or structure of SLA is necessary for WNV NS5 recognition. We thus determined the shapes of WNV and JEV SLAs, and WNV NS5 interactions with WNV, DENV, ZIKV, and JEV SLAs. WNV NS5 interacts with DENV, ZIKV, and JEV SLAs, yet this interaction does not always lead to viral replication. We next sought to determine the regions of WNV SLA that are important for WNV NS5 recognition and replication. The top loop and side loop sequences of WNV SLA are required for WNV NS5 interaction. Interestingly, a small reduction in binding to the WNV NS5 results in a drastic reduction of genomic replication. Finally, we show that exogenous SLA competes with genomic SLA during replication, likely by binding to WNV NS5, and functions as an inhibitor, resulting in reduced replication and viral infection. RNA sequencing of the recovered viruses showed an increase in single nucleotide variant (SNV) frequencies, indicative of selective pressure.

## RESULTS

### WNV and JEV SLAs have similar folds to other orthoflavivirus SLA

Despite differences in vector species and vertebrate amplification hosts, the structure and essential role of orthoflavivirus SLAs in viral replication are highly conserved (Fig. 1A) (25, 35). The SLA structures of isolated DENV and ZIKV SLAs and in complex with DENV NS5 show that SLA consists of top stem-loop, side loop, and bottom stem, yet their relative arrangements of top stem to bottom stem are varied (see Fig. 1D) (31, 33). Previous studies have shown that the WNV genome containing the 5′ UTR of DENV2 replicates as the corresponding WT, indicating that the WNV and DENV2 SLA can be interchangeable (36). However, NS5 protection assays of WNV and DENV showed that different regions of SLAs were protected, suggesting that WNV and DENV SLA may interact differently with NS5 (29, 37). Since the secondary structure prediction of WNV SLA shows a more open side loop than DENV, WNV SLA may fold into a different conformation from those seen in DENV and ZIKV SLAs (Fig. 1A). Since the SLA structures of WNV and JEV are lacking, we investigated the global fold of these SLAs using small-angle X-ray scattering (SAXS).

To stabilize the SLA structure, the WNV and JEV SLAs were expressed with a tRNA scaffold (SLA-tRNA), where the SLA sequence replaced the anticodon loop of human lysine tRNA (Fig. S1A) (38). This chimeric RNA design was previously used to determine the crystal structures and binding interactions of DENV2 and ZIKV SLAs (31). Scattering profiles of WNV and JEV SLAs were obtained at three concentrations, and the scattering curves of 1 mg/mL were used for further analysis (Fig. 1B and C). The molecular weights of WNV and JEV SLA-tRNAs were calculated using Porod volume to be 40–42 and 45–46 kDa, respectively, consistent with the molecular weights of SLA-tRNA monomers (46.5 and 45.8 kDa, respectively). Guinier plots, Kratky plot, and pair distance distribution of the WNV and JEV SLA-tRNAs indicated that they are similar in size and shape (Fig. 1B and C; Fig. S2A) (39). The radius of gyration (*R*_*g*_) of WNV and JEV SLA-tRNA are 40 and 39 Å, and the maximum diameter (*D*_max_) values are 127 and 121 Å, respectively.

We next calculated a three-dimensional (3D) shape for WNV and JEV SLA-tRNAs. Similar to the structure of DENV SLA-tRNA, both RNAs show a dumbbell-shaped envelope, where the top stem and side loop of SLA and the tRNA are connected by a long stem (Fig. 1C and E). To gain further insight, we modeled the 3D structures of WNV and JEV SLA-tRNA using AlphaFold 3 (AF3) (Fig. S2B) and compared their calculated scattering curves with the experimental ones (40). However, the WNV and JEV models fit the scattering profiles poorly with the χ^2^ values of 29.7 and 13.8, respectively. Thus, additional 3D models were generated using SCOPER, where an ensemble of molecular models was created from the AF3 model using molecular dynamics simulations, and their calculated scattering curves were compared to the experimental curves (41). The resulting 3D models of WNV and JEV SLA-tRNA fit the scattering curves with χ^2^ of 4.0 and 1.26, respectively (Fig. 1B and C; Fig. S2B and C). The final WNV and JEV SLA models form an “L”-shaped molecule, consisting of top, side, and bottom stems, roughly 85 Å long, similar to those of DENV2 and ZIKV (Fig. 1F) (31). However, the stacked top and side stem-loops in WNV and JEV SLA are differentially related to their bottom stem by 120°–130° (Fig. 1F).

### WNV NS5 and SLA form a 1:1 complex with a binding affinity similar to those determined for other orthoflaviviruses

**Fig 2:**
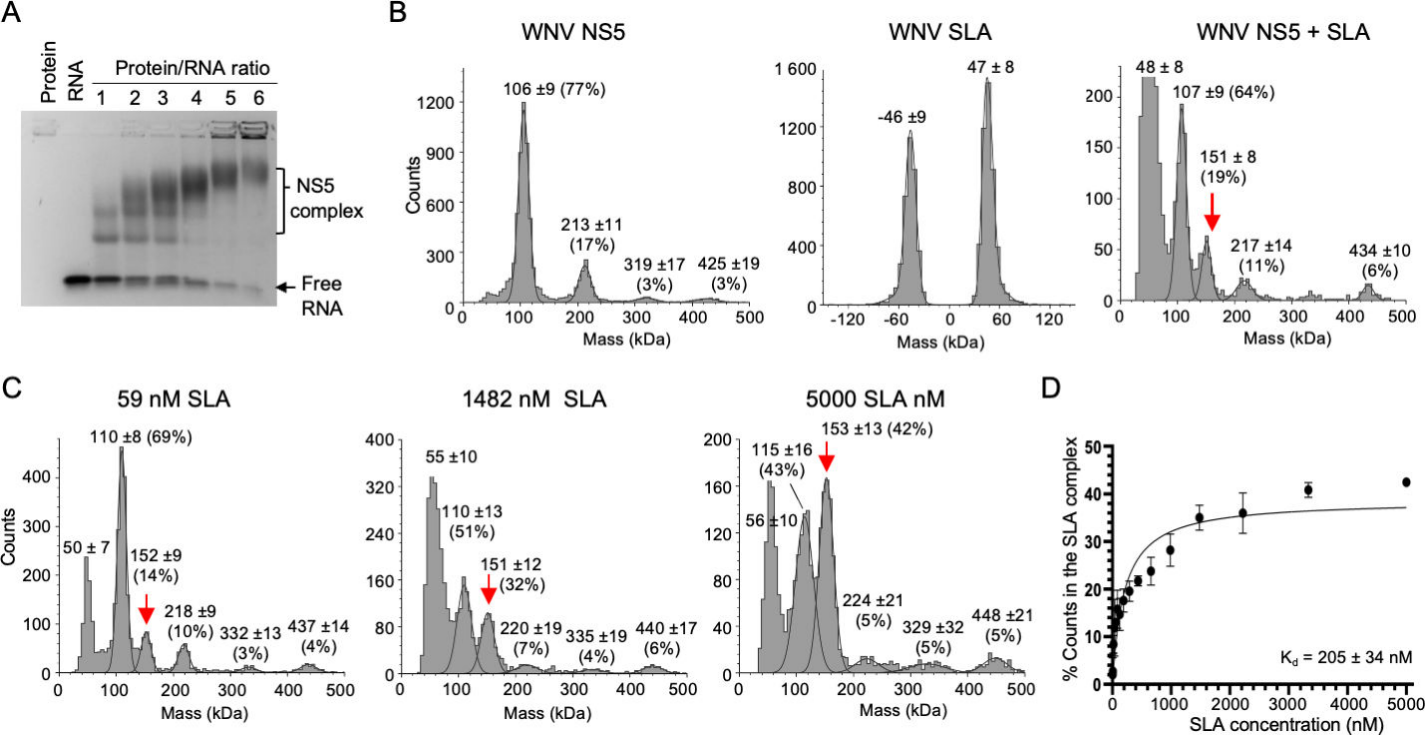
WNV NS5 interaction with WNV SLA. (**A**) Electrophoretic mobility shift assay. The WNV SLA-tRNA (1 µM) was titrated with increasing concentrations of WNV NS5 from 1 to 6 µM. EMSA was performed three times with similar results. (**B**) Mass photometry of the WNV NS5, SLA, and the NS5-SLA complex. WNV NS5 (20 nM), WNV SLA-tRNA (500 nM), and their mixture were analyzed by MP. The relative abundance of NS5 species is indicated in parentheses. WNV NS5 exists as a monomer (106 kDa), dimer, trimer, and tetramer. WNV SLA-tRNA shows a monomer peak (46 kDa) on the negative and positive spectra, indicating that the RNA is not settling on the slide. When incubated together, WNV NS5 forms a 1:1 complex with WNV SLA (152 kDa, red arrow), constituting 19% of total counts. (**C**) Titration of WNV NS5 with SLA. WNV NS5 (20 nM) was titrated with increasing concentrations of WNV SLA (3.4 nM to 5.0 µM), and the binding curve was created from the percentage counts of the NS5-SLA complex (**D**). The MP measurements were performed in triplicate for each RNA concentration. Three representative MP profiles are shown. As the SLA concentration increases, the percentage of counts corresponding to the NS5-SLA complex increases. (**D**) Binding curve of WNV NS5 with SLA. The binding curve was fit with the single binding site model using GraphPad Prism Software (version 10.2.3), and the *K*_*d*_ was determined to be 205 ± 34 nM.

WNV NS5 and SLA interaction was first tested using electrophoretic mobility shift assay (EMSA) using WNV SLA-tRNA. Incubation of SLA-tRNA with increasing amounts of WNV NS5 (one- to sixfold excess) led to multiple shifted bands, representing RNA-protein complexes (Fig. 2A). To ensure that NS5 binds the SLA portion of SLA-tRNA and not the tRNA, a competition EMSA with SLA-tRNA using tRNA as a competitor was also performed (Fig. S1B). The EMSA shows that NS5 selectively binds to SLA-tRNA over tRNA, as the SLA-tRNA band decreased with increasing NS5 concentrations, while the tRNA band remained unchanged. The formation of multiple complexes between NS5 and SLA has also been shown for DENV and ZIKV (31). Several orthoflavivirus NS5 structures have been determined as a dimer (20). Furthermore, DENV SLA forms a dimer both in solution and the crystal structure, with dimerization mediated by kissing loop interactions between the side loops (31). Thus, the multiple bands in EMSA may represent WNV NS5 monomer/dimer and SLA monomer/dimer complexes. Therefore, we examined the oligomeric states of WNV NS5 and SLA, and their interactions in solution using mass photometry (MP). In MP, the light scattered by a molecule on a glass plate interferes with light reflected by the surface. This interference signal correlates directly with molecular mass and could be used to determine stoichiometry and relative abundance of protein-RNA complexes (42, 43). When the full-length NS5 (20 nM) was used, four peaks were observed at 106 ± 9, 213 ± 11, 319 ± 17, and 425 ± 19 kDa (Fig. 2B). Since the calculated molecular mass of WNV NS5 is 106 kDa, we interpret that these peaks correspond to the NS5 monomer, dimer, trimer, and tetramer, respectively. The monomer and dimer were most abundant, with 77% and 17% of the total counts, respectively, and trimer and tetramer each accounted for ~3% of the total counts. When WNV SLA-tRNA (500 nM) was used in MP, a peak at 47 ± 8 kDa was observed (Fig. 2B). Since the calculated mass of SLA-tRNA is 46 kDa, SLA exists as a monomer in solution and does not form a dimer. An additional peak at −46 ± 9 kDa was also observed, which indicates that the RNA does not settle on the slide (42, 44). When the NS5 (20 nM) was incubated with SLA-tRNA (500 nM), a new peak was observed at 151 ± 9 kDa accounting for 20% of total counts on the average (from at least three measurements), in addition to the NS5 monomer, dimer, trimer, and tetramer peaks (Fig. 2B). The size (151 kDa) matches the molecular mass of the NS5 complex consisting of an NS5 monomer and an SLA molecule (106 + 46 = 152 kDa), indicating that NS5 forms a 1:1 complex with SLA.

We next determined the binding affinity of WNV NS5 with SLA-tRNA by mixing the NS5 (20 nM) with increasing concentrations of WNV SLA-tRNA (3.4 nM to 5.0 µM) (Fig. 2C). The percentage of the NS5-SLA complex at each SLA concentration was used to plot the binding curve, and the dissociation constant (*K*_*d*_) was determined to be 205 ± 34 nM by fitting the curve with a one-site binding model, where [NS5] + [SLA] ↔ [NS5][SLA] (Fig. 2D). Previously, DENV and ZIKV NS5 interactions with their corresponding SLAs were determined using fluorescence titration with a *K*_*d*_ of 181 nM for DENV and 280 nM for ZIKV (45, 46). Thus, the *K*_*d*_ value for WNV NS5 and SLA interaction is similar to those determined for DENV and ZIKV.

### WNV NS5 interacts with orthoflavivirus SLAs, but only DENV and ZIKV SLA can replace WNV SLA in the replicon assay

**Fig 3:**
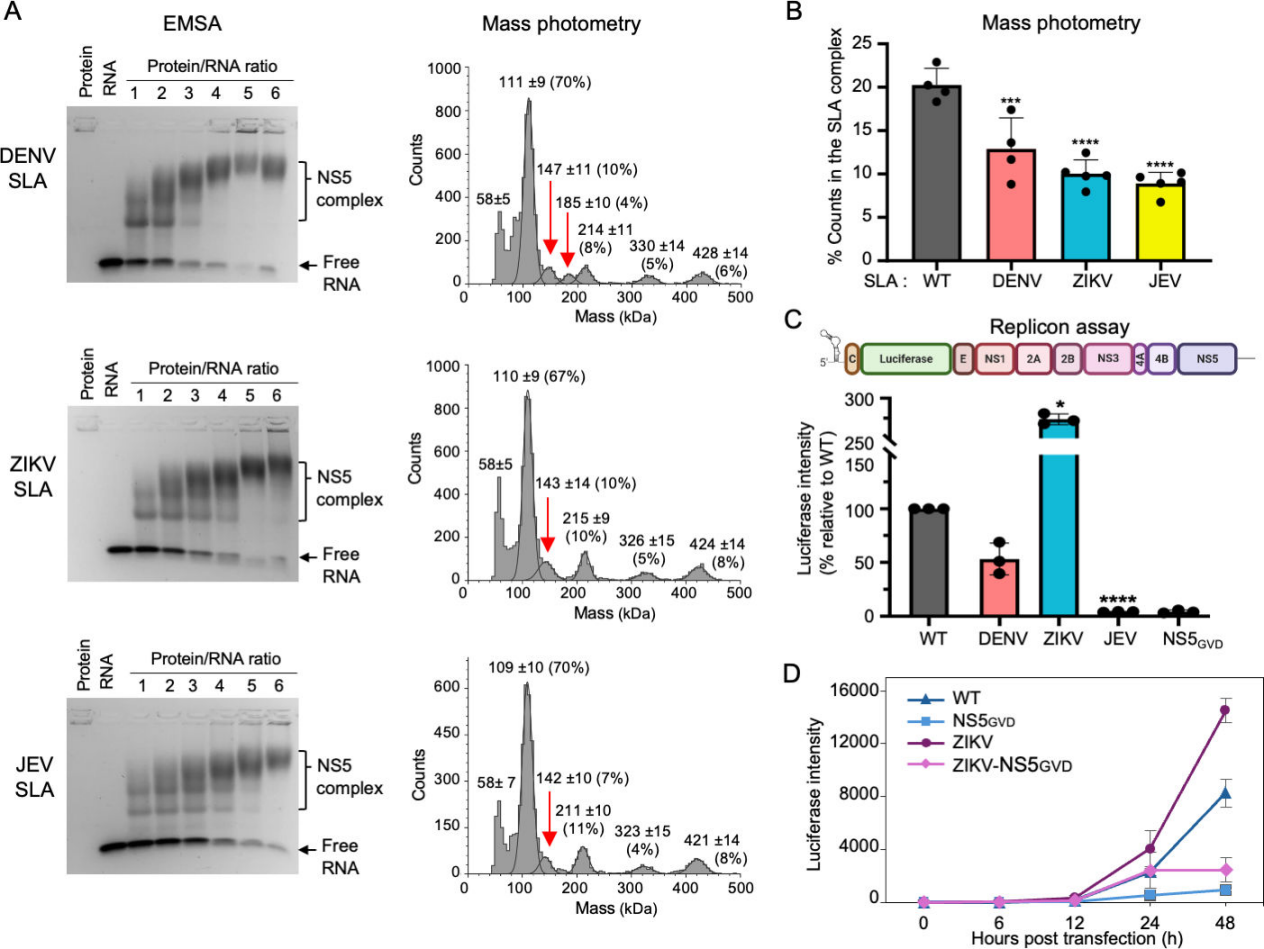
WNV NS5 interactions with orthoflavivirus SLAs. (**A**) Interaction between WNV NS5 and orthoflavivirus SLAs examined by EMSA (left) and mass photometry (right). In EMSA, DENV, ZIKV, and JEV SLA-tRNA (1 µM) were incubated with increasing concentrations of WNV NS5 (1–6 µM). WNV NS5 forms complexes with DENV, ZIKV, and JEV SLAs. Quantification of free RNA in EMSA gels is shown in Fig. S3A. In MP, WNV NS5 (20 nM) was incubated with orthoflavivirus SLA-tRNA (500 nM). NS5 binds orthoflavivirus SLA at 1:1 stoichiometry (151 kDa). The percentages of each NS5 species are indicated in parentheses, and the NS5-SLA complexes are indicated with red arrows. DENV SLA can form both monomer and dimer in solution (Fig. S3C) and forms an additional 2:1 complex of SLA:NS5 (calculated molecular mass of 196 kDa). (**B**) Summary of MP analysis. MP data were measured at least three times, and the averages of the percentage of counts corresponding to the NS5-SLA complex are shown. DENV2, ZIKV, and JEV SLAs had 1.6-, 2.0-, and 2.3-fold reduction compared to the WNV SLA, respectively. (**C**) WNV replicon assay containing orthoflavivirus SLAs. The WNV replicon contains the 5′ and 3′ UTRs as well as all NS proteins, while the structural genes are replaced with a luciferase gene (top). The SLA in the WNV replicon was replaced with that of DENV2, ZIKV, or JEV, and their effects on viral replication were measured by luciferase activity. Luciferase levels were normalized to the WT WNV replicon. Replacing the WNV SLA with DENV2, ZIKV, or JEV SLA changes luciferase levels to 53%, 273%, and 4% of WT. Asterisks indicate significant differences (*P < 0.005, **P < 0.001, ***P < 0.0005, and ****P < 0.0001) as determined by repeated measures one-way ANOVA followed by Dunnett’s multiple comparisons test in GraphPad Prism software (version 10.2.3). (**D**) Comparison of luciferase signal accumulation between WNV and ZIKV SLA-containing replicons. To determine whether the increased luciferase signal observed in the ZIKV SLA-containing replicon was due to enhanced translation, the WNV SLA on the replicon-incompetent NS5_GVD_ vector was replaced with ZIKV SLA (ZIKV-NS5_GVD_). WNV replicons containing either the WNV or ZIKV SLA, with or without the defective NS5 (WT, NS5 NS5_GVD_, ZIKV, and ZIKV-NS5_GVD_), were used to transfect BHK cells, and luciferase intensity was measured at various time points post-transfection. A faster rate of luciferase signal accumulation in ZIKV-NS5_GVD_ compared to WNV-NS5_GVD_ at early time points (<24 h) indicates that the increased signal is due, at least in part, to an NS5-independent mechanism, likely enhanced translation.

We next determined if WNV NS5 similarly interacts with other orthoflavivirus SLAs that are evolutionarily related to WNV and DENV. SLAs from DENV2, ZIKV, and JEV were constructed with the tRNA scaffold (Fig. S1A), and their interactions with WNV NS5 were tested by EMSA and MP (31, 38). In EMSA, WNV NS5 bound individual DENV2, ZIKV, and JEV SLAs and showed multiple shifted bands, similar to those observed with WNV SLA (Fig. 3A). Based on EMSA, WNV NS5 interaction with DENV and ZIKV SLAs was similar to WNV, while interaction with JEV SLA was reduced ( Fig. S3A).

We next measured WNV NS5 interactions with orthoflavivirus SLAs in solution using MP (Fig. 3A). All measurements were performed at least three times, and the averages are shown in Fig. 3B. We first tested if orthoflavivirus SLAs form a dimer in solution (Fig. S3C). Only DENV2 SLA showed both monomer and dimer peaks, consistent with the previous report (31). Consequently, WNV NS5 formed two types of the protein-RNA complexes in the presence of DENV2 SLA, one containing one NS5 and one SLA molecule (147 ± 10 kDa, heterodimer), and the other containing one NS5 and two SLA molecules (185 ± 10 kDa, heterotrimer) (Fig. 3A). Together, the WNV NS5 and DENV2 SLA complexes account for 14% of the total counts on the average. Incubation of WNV NS5 with ZIKV and JEV SLA led to a further reduction of 10% and 7% of the ribonucleoprotein complex formation (Fig. 3A and B). Thus, WNV NS5 binds all orthoflavivirus SLAs tested, representing three different complexes of the orthoflavivirus genus, although less efficiently than WNV SLA (20%, Fig. 2B).

While the amount of observed RNA–protein complex correlates with their binding affinities, the magnitude of reduction is not directly proportional to the decrease in binding affinity. Thus, we next tested if the interaction of WNV NS5 with orthoflavivirus SLA can lead to WNV replication. WNV luciferase-expressing replicon system, consisting of 5′ UTR, a luciferase gene, viral NS protein encoding genes, and 3′ UTR, was used (Fig. 3C) (47). We individually replaced the SLA in the WNV replicon with DENV2, ZIKV, or JEV SLA and measured luciferase activity at 48 h post-transfection (hpt). A replication-incompetent vector containing the NS5 D664V mutation, rendering the polymerase inactive (catalytic GDD to GVD), was used as a negative control (NS5_GVD_). Replacing the WNV SLA with DENV2 SLA led to a reduction in luciferase levels to 40% (Fig. 3C). Interestingly, WNV replicon containing JEV SLA had reduced luciferase levels to 4.2%, similar to the negative control, NS5_GVD_. Thus, JEV SLA could not functionally replace WNV SLA.

Interestingly, the WNV replicon containing the ZIKV SLA exhibited a 2.7-fold increase in luciferase signal (Fig. 3C). This increased luciferase signal could result from increased genome replication, enhanced RNA translation, or improved RNA stability. To distinguish among these possibilities, we constructed an additional WNV replicon harboring both the ZIKV SLA and the NS5 D664V mutation (ZIKV–NS5_GVD_). Luciferase activity was measured at 6, 12, 24, and 48 hpt for replicons containing either the WNV or ZIKV SLA (WT, NS5_GVD_, ZIKV, and ZIKV–NS5_GVD_) (Fig. 3D). Luciferase signals were detectable as early as 12 hpt. Consistent with prior results (Fig. 3C), replicons containing the ZIKV SLA (ZIKV and ZIKV–NS5_GVD_) showed higher luciferase activity compared to those with the WNV SLA (WT and NS5_GVD_). These findings suggest that the ZIKV SLA confers a translational advantage at early time points (<24 hpt) and a replication advantage at later time points (>24 hpt), contributing to the overall increased signal. Additionally, the kinetic data suggest that enhanced RNA stability is not a contributing factor, since the luciferase signals from NS5_GVD_ and ZIKV–NS5_GVD_ replicons declined at similar rates (Fig. 3D). Together, these data indicate that replacing the WNV SLA with the ZIKV SLA facilitates more efficient translation and replication of the replicon RNA.

### WNV SLA mutations in the top loop and side loop reduce NS5 interaction and viral replication

**Fig 4:**
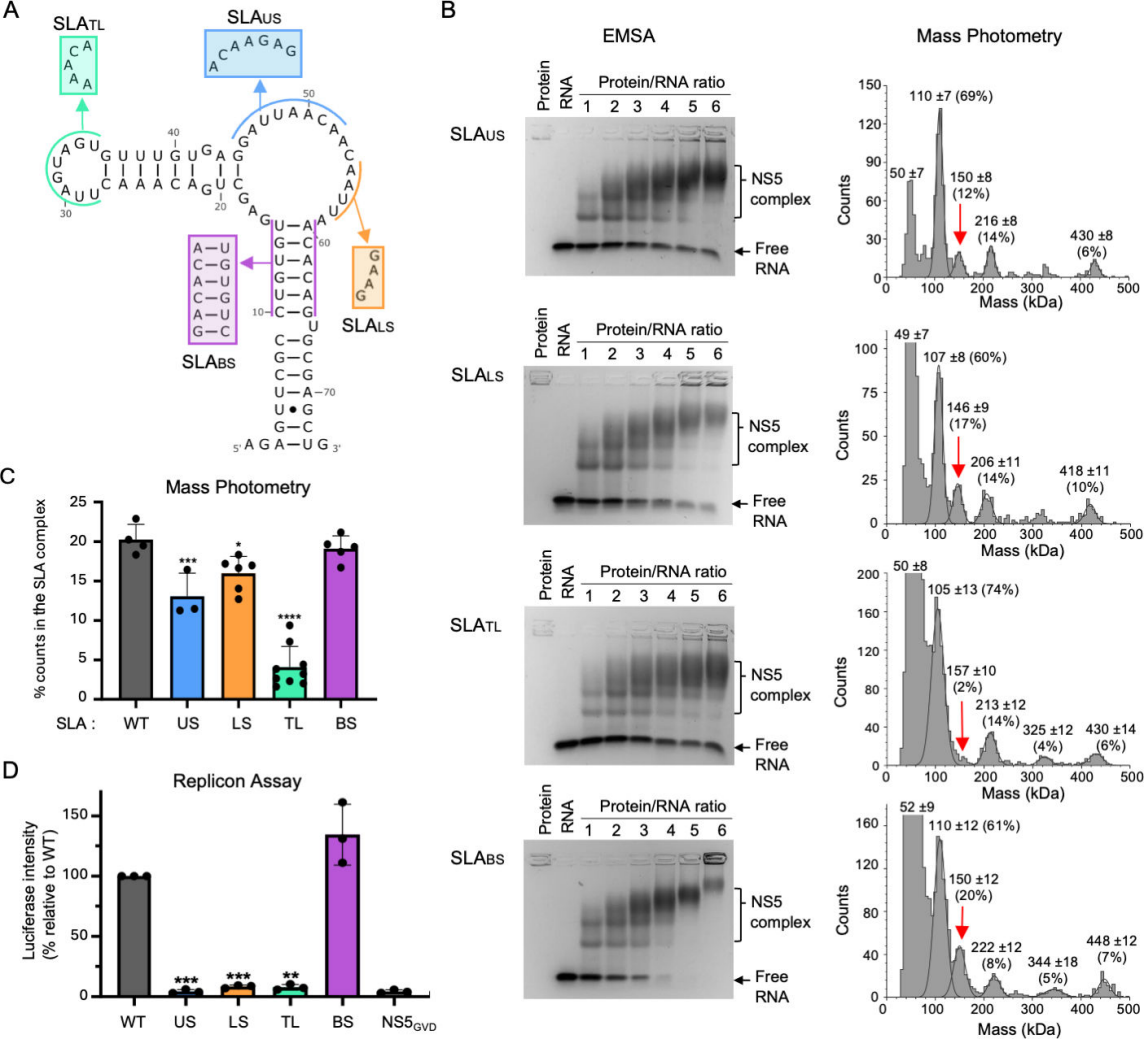
WNV NS5 interactions with WNV SLA mutants. (**A**) Design of WNV SLA mutants. SLA mutations are introduced to the top loop (SLA_TL_), upper side loop (SLA_US_), lower side loop (SLA_LS_), and the bottom stem (SLA_BS_) (boxed area). (**B**) WNV NS5 interaction with WNV SLA mutants examined by EMSA and MP. In EMSA, SLA mutants form multiple complexes with WNV NS5 to varying degrees (left). SLA_US_ and SLA_TL_ show greater reduction in NS5 binding than SLA_LS_ and SLA_BS_. Quantification of free RNA bands is shown in Fig. S3B. In MP, percentages of each NS5 peak (monomer, dimer, trimer, tetramer, and the SLA complex) are shown, and the peak corresponding to the NS5-SLA complex is indicated with a red arrow. Mutations in the top and side loop (SLA_US_, SLA_LS_, and SLA_TL_) led to significant reductions in binding to WNV NS5 compared to the WT SLA. (**C**) Summary of MP analysis. Percentages of the WNV NS5 and SLA complex were determined from three replicates. SLA_US_, SLA_LS_, and SLA_TL_ had 1.5-, 1.3-, and 4.4-fold reduction compared to the WNV SLA, respectively. (**D**) Replicon assay containing WNV SLA mutations. Viral replication was measured using a WNV replicon containing luciferase. Luciferase intensity was normalized to the WT replicon. A replication-incompetent replicon containing the NS5 GVD mutant was used as a negative control. SLA_TL_, SLA_US_, and SLA_LS_ reduced luciferase levels to 6%, 9%, and 10% of WT replicon. Asterisks indicate significant differences (*P < 0.005, **P < 0.001, ***P < 0.0005, and ****P < 0.0001) as detected by repeated measures one-way ANOVA, followed by Dunnett’s multiple comparisons test in GraphPad Prism software (version 10.2.3).

To identify WNV SLA regions important for WNV NS5 interaction, mutations were introduced into individual SLA domains, the bottom stem, top loop, and side loop, based on previous WNV SLA RNA protection and orthoflavivirus SLA binding assays (33, 37). The most conserved regions of orthoflavivirus SLA sequences are the AG dinucleotide in the top loop (positions 31–32) and the bottom stem sequence (Fig. 1A). Thus, in the top loop mutant (SLA_TL_), the AG sequence was removed from the top loop (^29^UUAGUAGU^36^) and replaced with a shorter AAACA (Fig. 4A). In the bottom stem mutant (SLA_BS_), the duplex region formed by nucleotides ^11^CUGUGU^16^ and ^61^ACACAG^66^ was substituted with GACACA and UGUGUC, respectively, to preserve base pairing. Additionally, two side loop mutants were designed to disrupt potential base-pairing interactions between nucleotides ^46^GAUU^49^ and ^56^AAUU^59^. These complementary sequences in the side loop are shown to form kissing loop interactions in DENV and ZIKV SLA (25). In the upper side loop mutant (SLA_US_), the ^46^GAUUAACA^53^ sequence was replaced with ACAAGAG. In the lower side loop mutant (SLA_LS_), the ^56^AAUU^59^ sequence was replaced with GAAG.

The fold of designed SLA mutants was predicted using the RNAfold web server to ensure that the sequence changes do not affect the predicted secondary structure of the SLA (32). EMSAs were performed to test if the mutant SLAs bind to WNV NS5. The mutant WNV SLAs were incubated with increasing amounts of WNV NS5 (one- to sixfold) (Fig. 4B). Similar to the WT SLA (SLA_WT_) (Fig. 2A), the mutant SLAs showed a decrease in free SLA with multiple shifted bands of RNA-protein complexes. Quantification of the free RNA bands indicated that the SLA_LS_ and SLA_TL_ mutants have reduced NS5 binding compared to the SLA_WT_ (Fig. S3B). Interestingly, NS5 showed slightly increased binding to the bottom stem mutant, SLA_BS_, compared to the SLA_WT_, suggesting that the bottom stem sequence of SLA is not important for NS5 interaction, provided the RNA duplex structure is maintained.

Next, MP was used to measure the relative binding affinities of WNV NS5 with the SLA mutants (Fig. 4B and C; Fig. S3D). WNV NS5 (20 nM) was incubated with SLA mutants (500 nM). Incubation with SLA_LS_ slightly reduced the abundance of the NS5-SLA complex to 16% on average (from at least three measurements), while incubation with WNV_US_ reduced the NS5-SLA complex further to 13%. SLA_TL_ had the greatest reduction in the NS5 complex formation with 4%. In comparison to the NS5 and SLA_WT_ complex forming 20% of the total counts (Fig. 2B), NS5 incubation with SLA_BS_ resulted in no significant change in the NS5-SLA complex with 19% of total counts. Thus, the binding affinities are highest with SLA_WT_, SLA_BS_ > SLA_LS_, SLA_US_ > SLA_TL_ (Fig. 4C). The results indicate that the WNV NS5 recognizes the SLA sequence of the top and side loops.

We next tested how the mutations to the SLA affect viral replication following the initial interaction with NS5 using a WNV luciferase-expressing replicon system (47). The four SLA mutations (Fig. 4A) were introduced into the SLA region within the WNV replicon. The replicons containing SLA_US_, SLA_LS_, and SLA_TL_ showed drastically diminished luciferase levels (Fig. 4D). The luciferase signal for SLA_US_ was at 4.3% of the WT, similar to the negative control, NS5_GVD_. The signal for SLA_LS_ and SLA_TL_ was just slightly above at 8.4% and 7.8% respectively, suggesting that these mutations in the top loop and side loop greatly reduced viral replication. Relative to WT, replicon containing SLA_BS_ had a similar level of replication (134% luciferase signal). Thus, the bottom stem mutant that has WT NS5 binding affinity also had no impact on viral replication.

### Exogenous SLA reduces viral replication and infection

**Fig 5:**
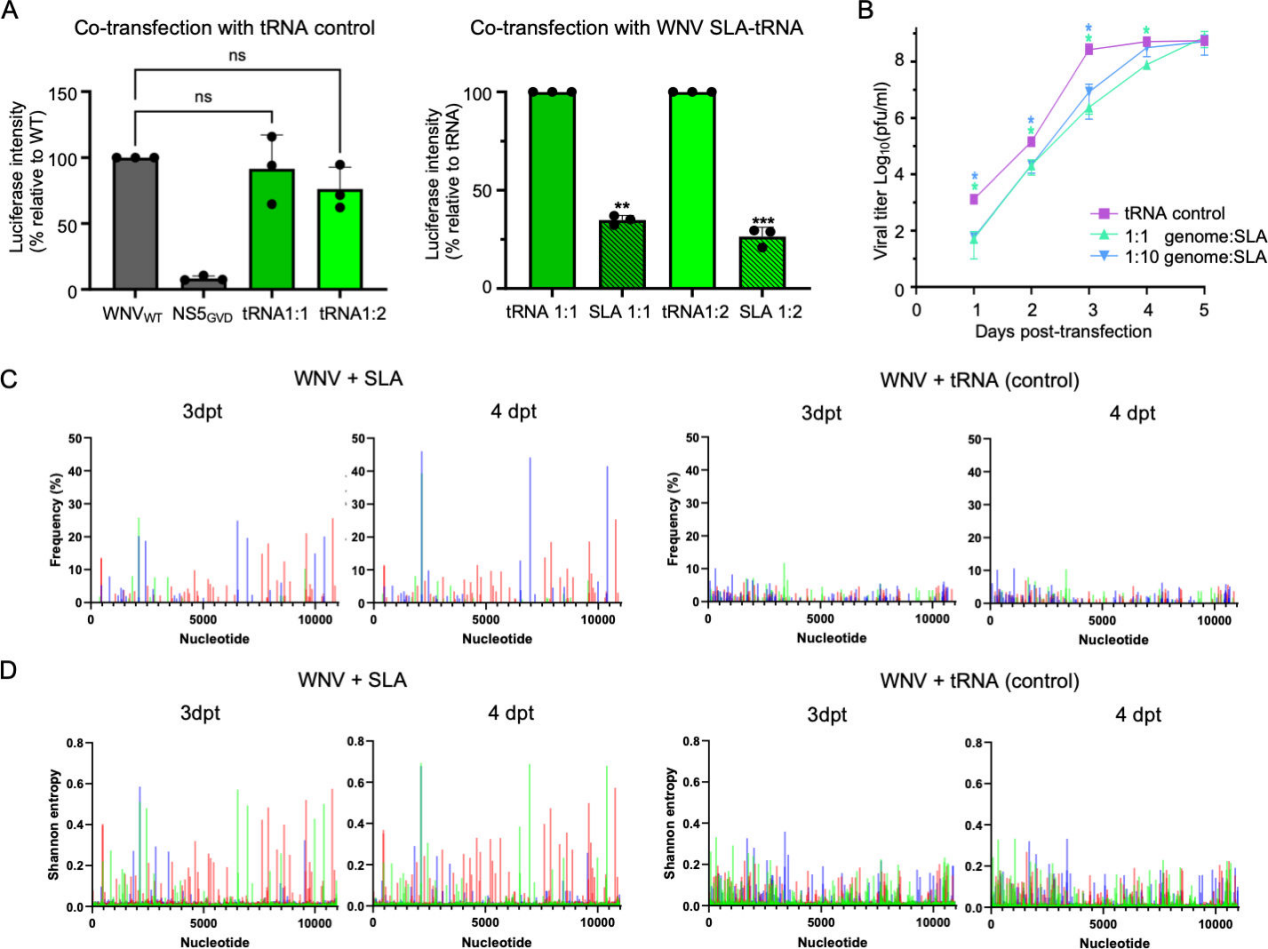
Exogenous SLA can inhibit WNV replication and viral infection. (**A**) Co-transfection of tRNA control and SLA-tRNA alongside WNV replicon. Replication is represented by luciferase intensity. Excess SLA significantly reduces luciferase levels in a dose-dependent manner, while co-transfection of tRNA with WNV replicon had no effect on the luciferase levels. Luciferase levels of SLA co-transfection were normalized to the tRNA control. Asterisks indicate significant differences (*P < 0.005, **P < 0.001, and ***P < 0.0005) compared to the tRNA control at the specific time point as determined by Student’s *t*-test using GraphPad Prism software (version 10.2.3). (**B**) Co-transfection of SLA-tRNA with WNV genomic RNA. Titers of recovered virus from cells transfected with the WNV genome and SLA-tRNA (or tRNA control) were determined using a plaque assay. Viral titer is significantly reduced 1–4 days post-transfection (dpt) in the presence of excess SLA during viral infection. Data points are mean ± standard deviation, *n* > 3. Asterisk (*) indicates significant difference (*P* < 0.005) compared to the tRNA control at the specific time point as determined by Student’s *t*-test using GraphPad Prism software (version 10.2.3). (**C**) Single nucleotide variant frequencies of the recovered viral genome. SNV frequencies of the WNV genome co-transfected alongside WNV SLA-tRNA or tRNA control were plotted for 3 and 4 dpt. Each color denotes a replicate. (**D**) Shannon entropy of recovered viral genome. Shannon entropy of WNV genome co-transfected alongside WNV SLA-tRNA or tRNA control was plotted for 3 and 4 dpt. Each color denotes a replicate.

WNV SLA interacts directly with WNV NS5 without additional cofactors from the replication complex (Fig. 2). Therefore, we hypothesized that the addition of exogenous SLA would compete against genomic SLA for NS5 interaction and reduce replication. We tested this idea by co-transfecting the WNV replicon with WNV SLA-tRNA at 1:1 or 1:2 (wt/wt) ratios. At a 1:1 (wt/wt) ratio, the luciferase signal was reduced to 35% of the tRNA control (Fig. 5A). At a 1:2 (wt/wt) ratio of the replicon and SLA-tRNA, the luciferase signal was further reduced to 26%, indicating that exogenous SLA acts as an inhibitor for replication, likely by depleting free NS5. As a control, co-transfection with tRNA at the same ratios had no effect on luciferase levels (Fig. 5A).

Since the presence of excess SLA can reduce replication of the WNV replicon, we next tested if exogenous SLA could diminish viral infection. We co-transfected WNV (lineage I) genomic RNA and WNV SLA-tRNA in equal and 10-fold (wt/wt) ratios. As a control, viral RNA was co-transfected with tRNA in an equal (wt/wt) ratio. When WNV genomic RNA and SLA-tRNA were co-transfected, viral titer was significantly reduced up to 4 days post-transfection (dpt) (Fig. 5B). Viral titer was reduced by ~10-fold for the first 2 dpt and reached a ~100-fold reduction on 3 dpt. Thus, exogenous SLA can act as an inhibitor for viral infection. The viral titer returned to control levels by 5 dpt, suggesting that exogenous SLA was consumed by binding to NS5 or degraded and no longer competes for viral replication. There was no significant difference in infectivity titer between equal or 10-fold excess (wt/wt) of WNV SLA.

### Next-generation sequencing indicates a role for SLA in orthoflavivirus viral RNA population diversity

To investigate genetic changes in WNV caused by the presence of exogenous SLA-tRNA, next-generation sequencing (NGS) analysis was performed on virus-containing cell culture supernatants in triplicate. Reads of both SLA-tRNA and tRNA co-transfection samples at 3 and 4 dpt were downsampled to 1,100 reads per nucleotide to match the sample with the lowest coverage. The mean depths of coverage at 1 and 2 dpt were <1,000 reads and thus were not analyzed. In general, the cells co-transfected with SLA-tRNA had fewer single nucleotide variants, but these occurred at higher frequencies than in cells co-transfected with tRNA (Fig. 5C). On 3 dpt, there were 75 unique and 1 repeat SNVs, with frequencies reaching 25.8% between the three replicates of the viruses recovered from the SLA co-transfected cells (Table 1). On 4 dpt, SNV frequencies reached 46.1% for 85 unique and 1 repeat SNVs. In comparison, the viruses from the tRNA-transfected cells had SNV frequencies reaching ~11.0% with 185 unique and 7–8 repeat SNVs on 3 and 4 dpt (Table 1).

**TABLE 1:** Instances of SNVs in recovered virus from the cells co-transfected with either SLA-tRNA or tRNA control

Co-transfection condition	Days post-transfection	Instances of SNVs at noted frequencies			Total SNVs
		1%–4.9%	5%–9.9%	>10%	
SLA	3	47	15	15	77
	4	54	20	13	87
tRNA control	3	180	17	2	199
	4	183	15	3	201

A total of 16 SNVs showed >10% frequencies from the SLA co-transfection (Table 2), 6 of which led to nonsynonymous substitutions in E, NS5, and 3′ UTR, i.e., SNVs were not random across the genome. E protein had two amino acid substitutions at E390G and Q391R. These residues reside within a highly conserved region of domain III and are shown to bind neutralizing antibodies (Fig. S4) (48). NS5 had two amino acid substitutions at T316S and K642R. The NS5 substitutions reside on the outer surface in the fingers and palm subdomains of the RdRp domain, respectively. T316S resides within an NS5 region that interacts with NS3 but does not itself interact with NS3 (Fig. S5) (33, 49). K642R is not located within the active site or RNA binding pocket. The last two SNVs were found in 3′ UTR at nucleotides 10,410 (A to T mutation) and 10,779 (A to C mutation). The 3′ UTR of WNV contains multiple stem-loop structures, including four stem-loops (SL-I to IV), two dumbbell-like structures (DB-1 and 2), and 3′SL (Fig. S6) (50, 51). The A10410 resides in the predicted double-strand stem of SL-I, while A10779 occurs within the top loop of the DB-I. The SL-I and DB structures are shown to be important to generate small flavivirus RNA and for viral pathogenesis (52). Comparatively, only one SNV was found for the viruses co-transfected with the tRNA control that led to a nonsynonymous amino acid substitution, K36Q in the E protein on 4 dpt (Table 2).

**TABLE 2:** SNV changes with greater than 10% frequencies in recovered virus from the cells co-transfected with either SLA-tRNA or tRNA control^*a*^

Co-transfection condition	Replicate	Days post-transfection	Frequency (%)	Nucleotide position	NT change		Protein	Synonymous	Non-synonymous
					From	To			
SLA	1	3, 4	20.2, 46.1	2135	A	G	E		E390G
		3	18.8	2436	A	T	E	G490	
		3, 4	24.9, 12.8	6528	A	T	NS4A	T20	
		3, 4	19.7, 44.1	6972	A	G	NS4B	Q19	
		3	14.9	9988	C	T	NS5	L770	
		3, 4	20.1, 41.5	10410			3′ UTR		A-T
	2	3, 4	13.6, 11.4	462	A	G	C	G122	
		3, 4	13.6, 11.2	465	A	C	C	A123	
		4	11.5	4614	A	T	NS3	G1	
		3, 4	14.8, 13.7	7623	A	C	NS4B	S236	
		3, 4	17.9, 18.5	7902	C	G	NS5	V74	
		3, 4	12.5, 10.4	8626	A	T	NS5		T316S
		3, 4	21.0, 18.6	9605	A	G	NS5		K642R
		3, 4	25.7, 25.4	10779			3′ UTR		A-C
	3	3, 4	25.8, 39.4	2138^*b*^	A	G	E		Q391R
		3	10.3	9552	G	A	NS5	R624	
tRNA (control)^*c*^	1	3, 4	10.1, 10.2	324	A	C	C	T76	
		4	10.6	1072	A	C	E		K36Q
	3	3, 4	11.7, 10.4	3384	A	G	NS1	G305	

^*a*^ Empty cells indicate “not applicable”.

^*b*^ A2138 to C change was also observed in SLA replicate 1 on days 3 and 4 at a low frequency (1.6% and 2.6%).

^*c*^ tRNA replicate 2 did not have any nucleotide changes over 10% frequency.

The NGS data were also analyzed using Shannon entropy, which is a measure of genomic heterogeneity (53). Shannon entropy was significantly higher for the viruses co-transfected with SLA-tRNA than the tRNA control, indicating that the presence of the SLA increased the heterogeneity of the viral RNA population compared to the tRNA control (Fig. 5D).

## DISCUSSION

The conservation of SLA and NS5 structures across orthoflaviviruses suggests that orthoflaviviruses utilize a similar replication mechanism (1, 25, 35, 36). However, the NS5 and SLA interactions predicted by footprinting assays differ drastically between WNV and DENV2 (29, 37). The WNV NS5 assay indicated that the upper side loop and bottom stem of WNV SLA are protected (37), while the DENV2 NS5 assay showed the top stem-loop and the lower side loop of DENV2 SLA are protected (29). These results suggest that the structure and NS5 interactions of SLA might be different between WNV and DENV. To better understand the structure and function relationship of orthoflavivirus SLA, we determined the molecular shapes of WNV as well as JEV SLAs using SAXS. Both WNV and JEV SLAs fold into an “L”-shaped molecule, similar to those of DENV and ZIKV SLAs (Fig. 1F). However, the relative arrangements of the top stem-loop and bottom stem seem to be different among orthoflaviviruses, suggesting preferred interactions with their cognate NS5.

We next tested WNV NS5 interaction with orthoflavivirus SLAs using EMSA and MP. WNV NS5 binds WNV SLA with the dissociation constant (*K*_*d*_) of 205 nM, comparable to the values obtained for DENV and ZIKV NS5 and SLA interactions, 181 and 280 nM, respectively (Fig. 2D) (45, 46). WNV NS5 was able to bind SLAs from other orthoflaviviruses (DENV2, ZIKV, and JEV) in both EMSA and MP binding assays. The binding affinities followed the order: WNV SLA (highest) > DENV2 SLA > ZIKV SLA > JEV SLA (lowest, with a 2.3-fold reduction). Accordingly, when orthoflavivirus SLAs were substituted into the WNV replicon in place of the native SLA, replication efficiency was generally reduced. The WNV replicon containing the DENV2 SLA produced approximately half the luciferase signal of the WT replicon, while the JEV SLA-containing replicon exhibited only baseline activity (Fig. 3C). These results indicate that relatively modest differences in WNV NS5–SLA interactions can lead to substantial differences in viral replication. The lack of replication with the JEV SLA was unexpected, given that WNV is phylogenetically more closely related to JEV than to DENV2, and that the WNV SLA shares similar sequence identity with DENV2 SLA (53%) and JEV SLA (52%) (Fig. 1A) (35).

Surprisingly, the ZIKV SLA-containing replicon exhibited a 2.7-fold increase in luciferase activity compared to the WT replicon (Fig. 3C), suggesting that the ZIKV SLA may confer additional replication advantages, possibly through enhanced translation. To test this, we conducted a time-course analysis of luciferase expression in WNV and ZIKV SLA-containing replicons. Our kinetic data (Fig. 3D) showed that the increased luciferase signal in the ZIKV SLA replicon persisted even when NS5 was catalytically inactive, indicating that the enhanced replication is at least partially driven by increased translation efficiency. It is possible that replacing the SLA introduced structural changes in the 5′ UTR that favor translation in the ZIKV SLA replicon.

Our results suggest that while NS5 and SLA interactions are necessary, they are not sufficient for efficient viral replication. Because the replication assay reflects multiple rounds of viral replication over a 48-h period, even modest reductions in NS5–SLA binding during the initial stages can lead to substantial decreases in overall replication. This may occur by disrupting downstream interactions with other components of the replication complex, as efficient viral replication likely also requires engagement of the NS5–SLA complex with additional components such as the NS3 protein or the 3′ UTR (28, 37). Orthoflaviviruses have a predicted stem-loop structure at the 3′ terminus of the genome (3′SL) that would interact with NS5 for viral RNA synthesis (22, 28). Thus, the WNV NS5 and JEV SLA complex may also impact its interaction with 3′SL due to differently arranged top and bottom stems. A future study could be directed to investigate how NS5 discriminates 5′SLA and 3′SL and how replacement of 3′SL alone and in conjunction with the 5′ SLA affects overall viral replication.

To identify the regions of WNV SLA that are recognized by the WNV NS5, SLA mutations in the top loop, side loop, and bottom stem were introduced. The top loop mutation in SLA showed the greatest reduction in NS5 binding by approximately fivefold, while the bottom stem mutant showed the WT binding in EMSA and MP assays. Similar to the results with orthoflavivirus SLAs, despite the relatively small changes of WNV NS5 interactions with top and side loop SLA mutants, WNV replicon containing the top and side loop SLA mutations showed significant reduction in viral replication or no viral replication (Fig. 4D). Thus, the sequences of the top and side loop of SLA are important for NS5 interaction. The recent cryo-EM structure of the DENV3 NS5 and SLA complex shows that the conserved AG in the top loop (^30^CAGU^33^) interacts with the thumb subdomain of the RdRp, while the side loop is exposed to the solvent (33). Thus, the top loop of WNV may similarly interact with NS5, consistent with our binding data. However, JEV SLA that have the identical “UAGU” sequence in the top loop did not replicate. Thus, NS5 interaction with the top loop is necessary but not sufficient for replication. Reduction in binding by the side loop mutants (SLA_US_ and SLA_LS_) could be explained by their role in SLA structures. The tertiary structures from DENV and ZIKV SLAs suggest that the side loop would be required to orient the top stem relative to the bottom stem. Thus, the side loop mutations could induce a structural change in SLA, ultimately impacting its ability to bind to NS5. This would be consistent with the observation that deletion of the side loop in DENV SLA abolishes viral replication, and deletion in WNV SLA greatly reduces viral replication despite the lack of sequence conservation in the side loop region (23, 26, 27).

### SLA can act as an inhibitor to viral replication

Orthoflavivirus NS5 has been a focus for developing therapeutics and RNA inhibitors due to its role in viral replication and inhibition of host type I interferon signaling (12, 19, 54, 55). Since the NS5-SLA interaction is essential for viral replication, we tested whether exogenous SLA could bind NS5 as an RNA decoy to inhibit replication and infection. Co-transfection of the WNV replicon with WNV SLA-tRNA showed that exogenous SLA reduced genomic replication in a dose-dependent manner (Fig. 5A). Furthermore, co-transfection of WNV genomic RNA and SLA-tRNA diminished active viral infection up to 4 dpt, indicating that the SLA can act as an inhibitor (Fig. 5B). The tRNA scaffold of SLA-tRNA likely contributes to its cellular stability and resistance to nucleases, similar to endogenous tRNAs. Typical tRNAs are known for their exceptionally long half-lives—estimated at 44 and 50 h in *Euglena gracilis* and chicken muscle, respectively (56). Structural studies of tRNA-scaffolded SLA have shown well-folded SLA and tRNA domains (31). Furthermore, the tRNA scaffold is likely post-transcriptionally modified, such as by methylation, which enhances stability by protecting against degradation (38). This extended stability likely underlies the observed inhibitory effect of SLA-tRNA.

As the quantity of viral genomes and NS5 proteins increases through multiple rounds of viral replication, the initial exogenous SLA is outcompeted for binding NS5 and possibly degraded, leading to a recovery in viral infection at day 5. The recovered virus sequences from the cells co-transfected with SLA had a greater genetic diversity compared to the tRNA control. While there was only one repeat SNV between three replicates in the viral genomes of cells co-transfected with the SLA, the higher frequency of SNVs is indicative of a genetic selection pressure, i.e., the selective pressure of SLA as an inhibitor likely allows genetic diversity to accumulate, as shown by the increase in SE and SNVs. Further studies would determine if these SNVs continue to emerge and how they impact the fitness of the virus to inform the feasibility of excess SLA as a viable therapeutic antiviral option.

## MATERIALS AND METHODS

### Construction, expression, and purification of WNV full-length NS5

Full-length lineage II WNV NS5 (GenPept NP_776022) was created from the WNV replicon plasmid, kindly provided by Dr. Brian Geiss (Colorado State University). The full-length NS5 was cloned into the pMCSG7 vector with an N-terminal His-tag. WNV full-length NS5 was expressed in BL21-CodonPlus-RIL *Escherichia coli* cells (Stratagene) and purified as previously described (20). Briefly, cells were grown in Luria Broth with 25 µg/mL chloramphenicol and 1 µg/mL ampicillin at 37°C until OD_600_ reached 0.8–1.0. Cells were then chilled on ice prior to inducing protein expression with 1 mM isopropyl β-D-1-thiogalactopyranoside and grown overnight at 18°C. Cells were pelleted and resuspended in lysis buffer (50 mM Tris-HCl, pH 7.4, 500 mM NaCl, 5 mM β-mercaptoethanol [BME], and one EDTA-free protease inhibitor cocktail tablet [Roche Applied Science]) prior to sonication. Proteins were purified using TALON metal affinity resin (Clontech) in elution buffer (50 mM Tris-HCl, pH 7.4, 500 mM NaCl, and 5 mM BME) with a gradient of 5–400 mM imidazole. Fractions containing full-length NS5 were concentrated to 1 mL (Amicon) and further purified by size exclusion chromatography using a HiLoad 16/60 Superdex 200 preparative grade column (GE Healthcare) in gel filtration buffer (50 mM Tris-HCl, pH 7.5, 300 mM NaCl, 5 mM BME, and 5% glycerol). Protein concentration was determined by absorbance at 280 nm with extinction coefficients of 221,730 M^−1^ cm^−1^. The purified NS5 protein was further concentrated to 1 mg/mL and stored until use.

### Construction, expression, and purification of orthoflaviviral SLAs and WNV SLA mutants

WNV (NBI accession number, NC_001563) and JEV SLA (NC_001437) constructs were created and designed using a tRNA scaffolding approach, described previously (31, 38, 57). Briefly, the sequence of the respective SLA was placed within the anticodon loop of human lysine tRNA so that the tRNA sequence flanks the SLA sequence. The resulting sequence was synthesized in DNA2.0 (Newark, CA, USA) and inserted into the pBluescript II SK vector. WNV SLA mutants were generated via multiple site-directed mutagenesis to the wild-type WNV SLA construct. The construction of DENV2 (NC_001474) and ZIKV (KU527068) SLA-tRNA constructs was previously described (31).

The SLA-tRNA constructs were expressed and purified as previously described (31, 38). Briefly, the plasmids containing the SLA-tRNA constructs were transformed into BL21 *E. coli* cells (Stratagene) and grown in 2× TY medium with 1 µg/mL ampicillin overnight at 37°C. The cells were pelleted and resuspended in 10 mM magnesium acetate and 10 mM Tris-HCl, pH 7.4. The RNA was purified via phenol extraction followed by ethanol precipitation. The resulting RNA pellet was resuspended in Buffer A (50 mM Tris-HCl, pH 7.4) and loaded on a HiLoad 16/60 Q ion-exchange column (GE Healthcare) for anion exchange chromatography. RNA was eluted with a 400–700 mM NaCl gradient in Buffer A. Fractions containing the RNA were pooled and dialyzed into the gel filtration buffer (50 mM Tris-HCl, pH 7.5, 300 mM NaCl, 5 mM BME, and 5% glycerol) using dialysis membrane (Spectra Por). Alternatively, the pooled RNA was buffer exchanged to water. RNA concentrations were determined by absorbance at 260 nm using the extinction coefficients determined from the sequence using an oligo extinction coefficient calculator of 40 (mg/mL)^−1^ cm^−1^ (https://www.fechem.uzh.ch/MT/links/ext.html)

### Small-angle X-ray scattering data acquisition and analysis

To determine the shape of WNV and JEV SLAs, SAXS data were acquired at the LiX beamline at Brookhaven National Laboratory. The SAXS data were collected at three RNA concentrations (1, 2, and 4 mg/mL) in buffer containing 50 mM HEPES, pH 7.5, 150 mM NaCl, and 3 mM MgCl_2_. The data were measured in 60 frames with an exposure time of 0.3–0.5 s per frame using a flow cell to minimize radiation damage. The 2D images were reduced to 1D scattering profiles, then averaged using the Matlab scripts developed by the beamline. The matching buffer was used in a background subtraction from the RNA solutions. The scattering profiles of three RNA concentrations were analyzed individually using PRIMUS in the ATSAS program package (http://www.embl-hamburg.de/biosaxs/) (39). A Guinier plot was generated as ln(*I(q*)) versus *q*^2^ to check sample quality and obtain *I_0_* and the radius of gyration (*R_g_*) within the range of *q*_max_*R_g_ < 1.3. The data of each RNA were normalized with *I_0_*. The conformation of each RNA was examined using a dimensionless Kratky plot for *q* < 0.3 Å^−1^. Scattering profiles of each RNA were then Fourier-transformed using GNOM of the ATSAS package to obtain the normalized pair distance distribution graph. To obtain the overall shape of the SLAs, the reference envelope of each RNA was first built using dammif, damaverage, damstart, and damfilt (58–60). Using the reference envelope model as a starting point, Dammin was used to generate the representative beads model. Additionally, AlphaFold 3 was used to generate the initial 3D models of WNV and JEV SLA-tRNAs, which were further refined using SCOPER (40, 41). SCOPER uses MD simulations to generate optimal conformations of the model with the lowest χ^2^ by comparing its MD models with the scattering data.

### NS5 and SLA-tRNA interaction by electrophoretic mobility shift assay

WNV NS5 protein interactions with various SLA were determined using electrophoretic mobility shift assays. Orthoflavivirus or WNV mutant SLA-tRNA (1 µM) was mixed with full-length NS5 in one- to sixfold molar excess. Protein and RNA were mixed in binding buffer (50 mM Tris-HCl, pH 7.5, 420 mM NaCl, 2 mM MnCl_2_, 1 mM TCEP, 0.5 mg/mL BSA, 4.5 mM BME, and 5% glycerol). After a 30-min incubation at room temperature, samples were loaded onto a 1.2% agarose gel and separated on ice at 100 V for 50 min. SYBR Safe DNA Gel Stain (Invitrogen) was added to the gel to visualize the RNA. The gels were also stained with Coomassie stain to confirm that the shifted RNA bands contained protein. EMSAs were repeated three or more times to confirm consistent results. Intensity of bands, RNA alone and complexed, was measured using ImageJ (61). The fraction of free RNA was determined for each lane and plotted in GraphPad Prism software (version 10.2.3).

### Mass photometry of the WNV NS5-SLA complex

The stoichiometry of WNV NS5 full-length and its SLA complexes was determined using TwoMP mass photometry (Refeyn Ltd.). WNV NS5 (20 nM) was mixed with SLA-tRNA (500 nM) in MP buffer (50 mM HEPES, pH 7.5, 40 mM NaCl, and 10 mM MgCl_2_). The binding curve for WNV NS5 and SLA was generated by mixing NS5 at 20 nM with SLA-tRNA from 3.4 nM to 5 µM concentrations. Data were acquired over 60 s using AquireMP software (Refeyn Ltd.) and processed using DiscoverMP software (Refeyn Ltd.). DENV NS3 and NS5 proteins were used as standards to calculate the molecular mass. Data were collected at least three times for each concentration, and the percentage of total counts that corresponded to the NS5-SLA complex was plotted using GraphPad Prism software (version 10.2.3). MP data for orthoflavivirus SLAs (DENV, ZIKV, and JEV) and WNV SLA mutants (SLA_US_, SLA_LS_, SLA_TL_, and SLA_BS_) were similarly determined using WNV NS5 (20 nM) and SLA-tRNA (500 nM) in MP buffer (50 mM HEPES, pH 7.5, 40 mM NaCl, and 10 mM MgCl_2_).

### Construction of WNV replicon mutants and replication assay

WNV lineage II wild-type and NS5_GVD_ mutant replicon plasmids were kindly provided by Dr. Brian Geiss (Colorado State University) (47). SLA mutations were introduced to the WNV replicon utilizing NEBuilder HiFi DNA Assembly Master Mix (New England Biolabs). Plasmids were transformed into 10-beta competent *E. coli* (New England Biolabs) and purified via Qiagen Plasmid Midi Kit (Qiagen). The WNV replication assay was performed in baby hamster kidney (BHK) cells as previously described (47). BHK cells were grown at 37°C and 5% CO_2_ in humidified incubators and maintained using GIBCO high-glucose DMEM supplemented with 10% fetal bovine serum (FBS), 5% penicillin/streptomycin, and 50 mM HEPES. Briefly, BHK cells were seeded at 100,000 cells/well in a 24-well plate and allowed to attach overnight. WNV replicon plasmids were transfected at 250 ng/well the next day using Lipofectamine 2000 (Life Technologies). For co-transfection studies, cells were co-transfected at 250 ng/well with the tRNA or SLA-tRNA at 250 or 500 ng/well for 1:1 and 1:2 (wt/wt), respectively. The cells were lysed at 48 h post-transfection and transferred to black-walled, clear-bottom 96-well plates for imaging following the Luciferase Assay System (Promega). For kinetic assays, cells were transfected with 500 ng/well of DNA to enhance signal detection at early time points. Cells were lysed at 0, 6, 12, 24, and 48 h post-transfection and analyzed for luciferase activity. Luminescence signal was measured with a BioTek Synergy H1 Microplate Reader (Agilent). Transfection assays were repeated at least three times, and the mean and standard deviations were calculated and plotted using GraphPad Prism software (version 10.2.3).

### WNV NY99ic plasmid preparation

WNV infectious clone is based on lineage I strain NY99 (NY99ic). It is a two-plasmid system with one plasmid containing the 5′ UTR and structural genes, and the other containing the non-structural genes along with the 3′ UTR. The WNV NY99ic viral RNA (vRNA) was prepared as previously described (62). Briefly, plasmids were transformed in XL10 Gold cells (Agilent) and purified using the Qiagen Maxiprep Kit (Qiagen). The individual plasmids were digested and ligated together prior to purification via the Zymo DNA Clean and Concentrator-5 kit. The purified DNA was transcribed using the Ampliscribe T7 Flash Transcription kit (Lucigen), and the resulting RNA was purified using the Zymo RNA Clean and Concentrator-5 kit. The concentration of the vRNA was determined by spectrophotometer and used for co-transfection alongside WNV SLA-tRNA.

### WNV genome and SLA-tRNA co-transfection

Vero monkey kidney (ATCC CCL-81) cells were grown at 37°C and 5% CO_2_ in humidified incubators. Vero cells were maintained in 1× minimum essential media (MEM) supplemented with 8% FBS, 100 units/mL penicillin, 100 µg/mL streptomycin, 2 mM L-glutamine, and 0.1 mM non-essential amino acids. To co-transfect the vRNA and SLA RNAs, RNA mixtures were prepared with 90 µg vRNA alongside 90 µg tRNA (1:1, wt/wt ratio) as a control, in addition to 90 µg (1:1) and 900 µg (1:10) SLA-tRNA. The RNA mixtures were added to 3.4 × 10^6^ Vero cells in Dulbecco’s phosphate-buffered saline (DPBS), electroporated with 1.5 kV, 25 µF, ∞ ohms using the Gene Pulser (Bio-Rad), and then allowed to rest at room temperature (RT) for 10 min. Cells were allowed to grow for up to 5 days post-transfection with aliquots of virus collected daily and stored at −80°C.

Plaque assays were performed to determine the infectivity titer of virus recovered from 1 to 5 dpt. Vero cells at 80%–90% confluency in 6-well cell culture plates were washed with DPBS. Tenfold serial dilutions of the recovered virus from 1 to 5 dpt were added to each well prior to incubating while rocking at RT for 30 min. Maintenance MEM containing 1% agarose was added to each well and incubated for 48 h. On day 3 post-infection, maintenance MEM containing 1% agarose along with 2% neutral red was added to each well and returned to incubation. The following day, plaques were counted, and infectivity titers of each well were determined.

### Next-generation sequencing and data analysis

Viral RNA was extracted from the thawed aliquots using the Qiagen Viral RNA Mini Kit following the manufacturer’s instructions. Sequencing libraries were constructed using random hexamer primers, and 75 nt, paired-end sequences were run on the Illumina NextSeq 550 instrument in the UTMB Sequencing Core. Reads were trimmed to a minimum length of 35 bases, with reads below a quality score of 30 removed using Trimmomatic (version 0.39). Reads were downsampled to match those with the lowest mean coverage using samtools (version 1.18). They were aligned to a WNV NY99 reference sequence (GenBank: AF196835.2) using bowtie2 (version 2.5.1) using the very-sensitive-local parameters. Reads were sorted by coordinate, and PCR duplicates were removed using Picard (version 2.18). Single nucleotide variants were identified using Lofreq, and those ≥1% frequency were kept for analysis (version 2.1.5). Shannon entropy was calculated in R using custom scripts over possible nucleotide variants (A, U, C, G, and –) utilizing the formula previously published by Nishijima et al (63). Statistical analysis of Shannon entropy between co-transfection conditions was performed using GraphPad Prism Software (version 10.2.3) (64). Briefly, the Kruskal-Wallis test with Dunn’s multiple comparisons test was used to identify statistical significance, where *P* < 0.05. RNA from days 1–4 post-transfection was sequenced; however, the mean depth of coverage at 1 and 2 dpt was <1,000 reads and thus was not analyzed.

## Data Availability

Raw sequence reads (fastq files) were deposited at BioStudies (https://www.ebi.ac.uk/biostudies/arrayexpress/studies/) with accession number E-MTAB-14867.

## SUPPLEMENTAL MATERIAL

Supplemental material jvi.02023-24-s0001.pdf Figures S1 to S6.

ASM does not own the copyrights to Supplemental Material that may be linked to, or accessed through, an article. The authors have granted ASM a non-exclusive, world-wide license to publish the Supplemental Material files. Please contact the corresponding author directly for reuse.
