# Supplementary material for: Interaction of West Nile virus NS5 with orthoflavivirus SLA RNAs and their effects on viral replication and inhibition
Source: J Virol. 2025 Aug 18;99(9):e02023-24. doi: 10.1128/jvi.02023-24 (PMC12455937; doi:10.1128/jvi.02023-24)
Supplement: Supplemental material — Figures S1 to S6. [file jvi.02023-24-s0001.pdf]

Supplemental Materials for

Interaction of West Nile virus NS5 with orthoflavivirus SLA RNAs and their effects on viral replication and inhibition

Mandi A. Feinberg[1,2], My T. Le[3], Kassandra Carpio[1], Ekaterina Knyazhanskaya[3], Alan D. T. Barrett[4], and Kyung H. Choi[1,2,3, *]

1. Department of Biochemistry and Molecular Biology, University of Texas Medical Branch, Galveston, TX 77555, USA
2. Sealy Center for Structural Biology, University of Texas Medical Branch, Galveston, TX 77555, USA
3. Department of Molecular and Cellular Biochemistry, Indiana University, Bloomington, IN 47405, USA
4. Department of Pathology, University of Texas Medical Branch, Galveston, TX 77555, USA

Running title: Viral polymerase NS5 and RNA promoter interactions in orthoflaviviruses

[*]To whom correspondence should be addressed. Email: kaychoi@iu.edu

Mandi A. Feinberg and My T. Le contributed equally to this work.

Kassandra Carpio, Ekaterina Knyazhanskaya contributed equally to this work

Fig S1-S6

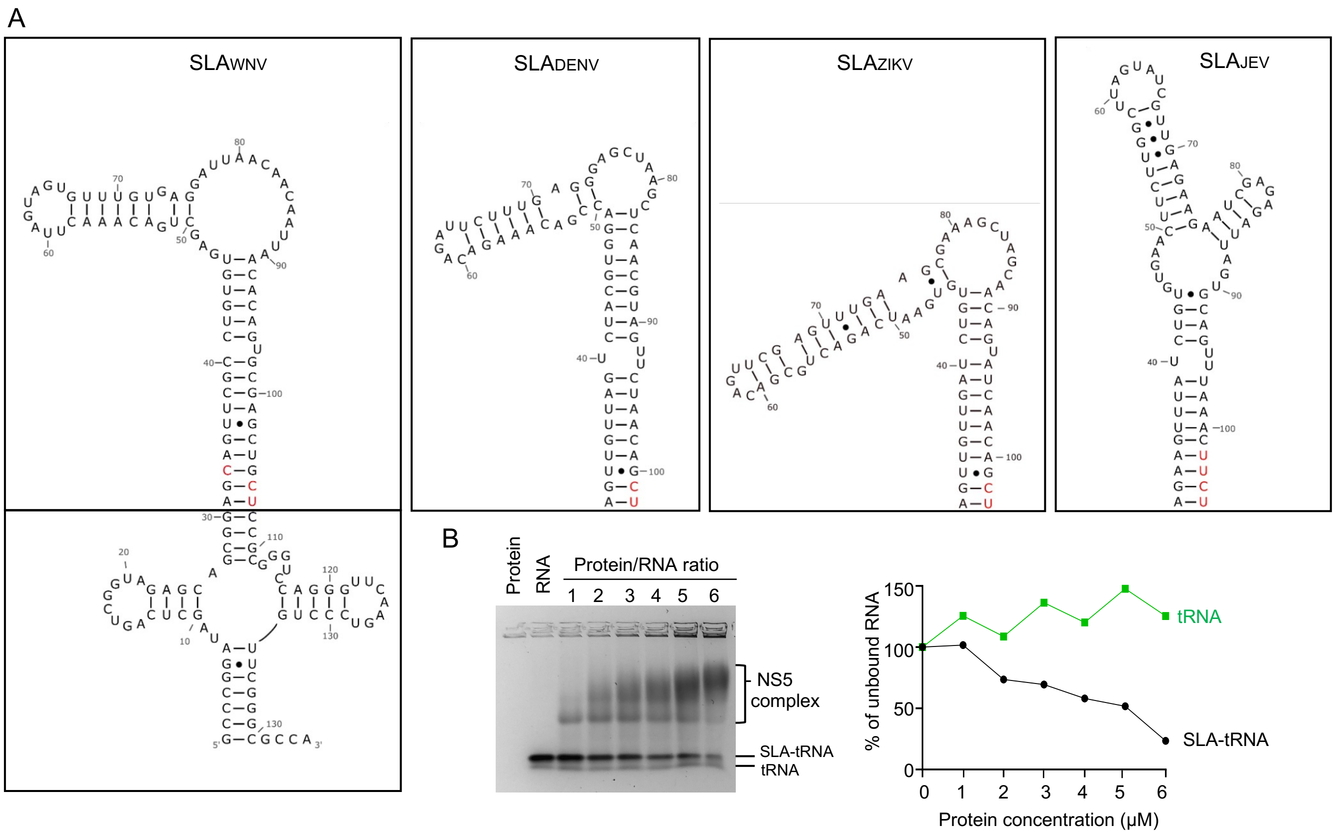

Fig S1. Design of orthoflavivirus SLA using tRNA scaffold. (A) The SLA-tRNA constructs used in the study. The regions of WNV, DENV2, ZIKV, and JEV SLA and tRNA are indicated. The red nucleotides were added to the SLA sequence to form base pairs. **(B)** Competition EMSA with tRNA and SLA-tRNA. WNV NS5 interaction with WNV SLA-tRNA was tested using tRNA as a competitor. The SLA-tRNA (1 μM) was titrated with increasing concentrations of WNV NS5 (1-6 fold excess) in the presence of tRNA (1 μM). The nucleic acids were stained with ethidium bromide and visualized under UV. Quantification of the unbound RNA bands is shown on the right. The SLA-tRNA bands decrease as the protein concentration increases, while the tRNA bands remain constant. The competition assay was repeated three times with similar results.

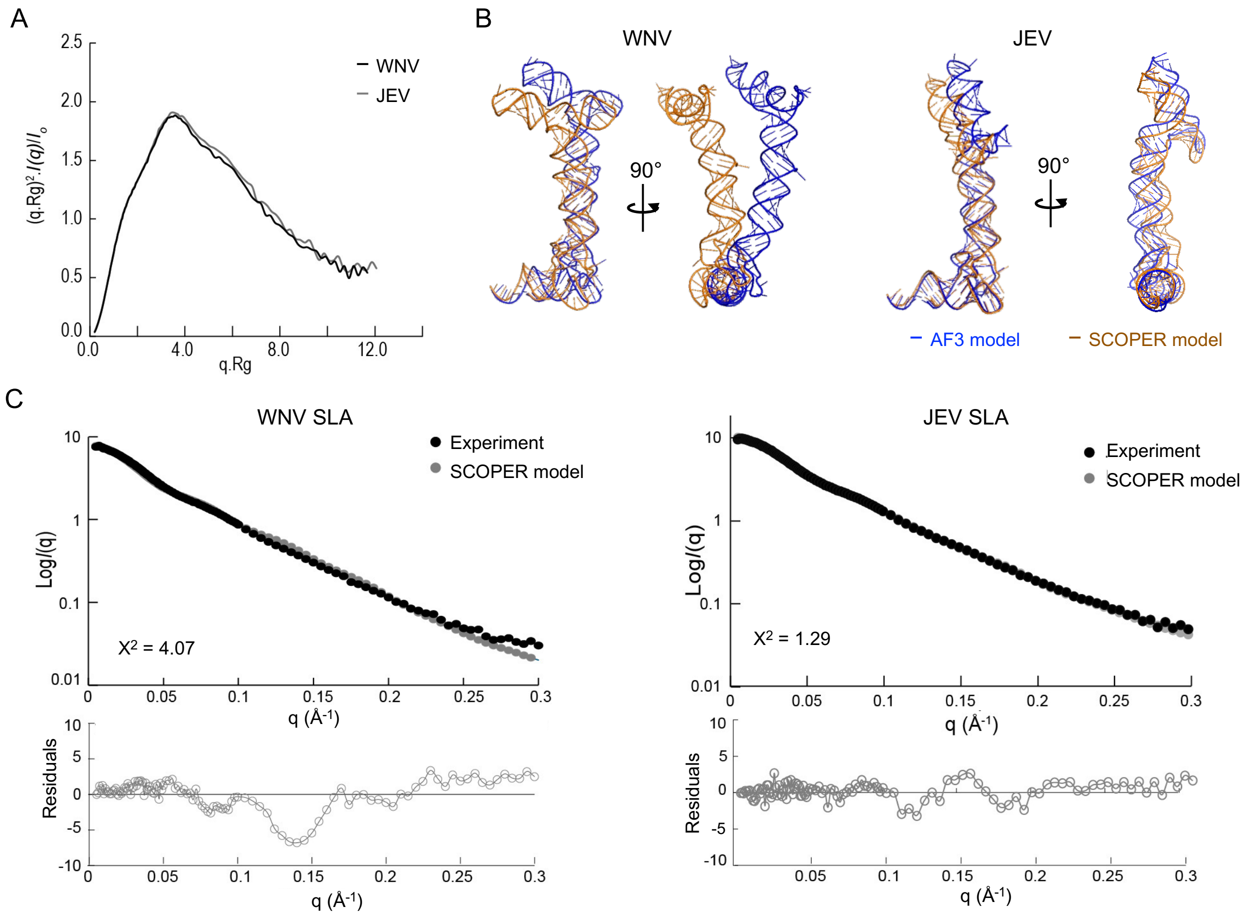

Fig S2. Small-angle X-ray scattering of WNV and JEV SLA-tRNA. **(A)** The Kratky plots of WNV and JEV SLA-tRNA. WNV and JEV SLA-tRNA are shown in black and gray, respectively. Both plots indicate well-folded RNA structures. **(B)** Modeling of WNV and JEV SLA-tRNA. The Alphafold3 models are shown in blue, and SCOPER models are shown in yellow. **(C)** The fit of SCOPER models (gray) to the experimental scattering curves (black) are shown. The residuals of the fit are shown at the bottom.

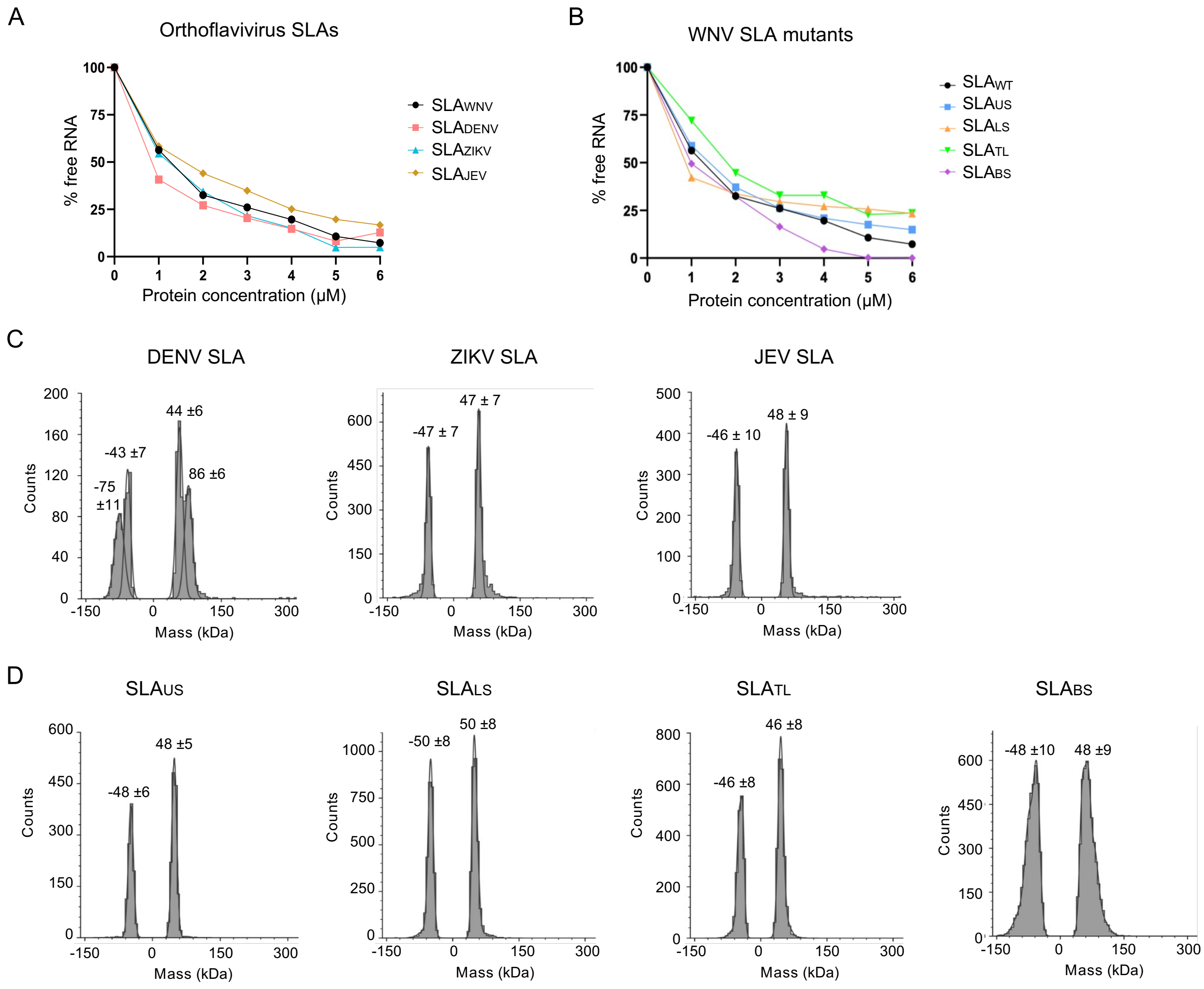

Fig S3. EMSA quantification and mass photometry of SLAs alone. (A) Quantification of free RNA in orthoflavivirus EMSAs. Band intensities for the EMSAs in fig 3A were measured using ImageJ.[3] The percent of free RNA was calculated and plotted in GraphPad Prism software (v10.2.3). WNV NS5 binds WNV, DENV2, and ZIKV SLA similarly, while it shows a reduced binding to JEV SLA. **(B)** Quantification of free RNA in WNV SLA mutant EMSAs. The band intensities in Fig 4B were similarly analyzed as in (A). WNV NS5 interaction with SLA_{TL} is most reduced among SLA mutants tested. SLA_{BS} has a slight increase in binding to WNV NS5. **(C)** **(D)** MP spectra of orthoflavivirus and WNV mutant SLAs. The concentration of the SLA solutions

is 500 nM. SLA showed a peak on both the negative and positive spectra indicating that the RNA is not settling on the slide. The calculated masses for DENV2, ZIKV and JEV are 44.9, 45.2, and 45.9 kDa, respectively.

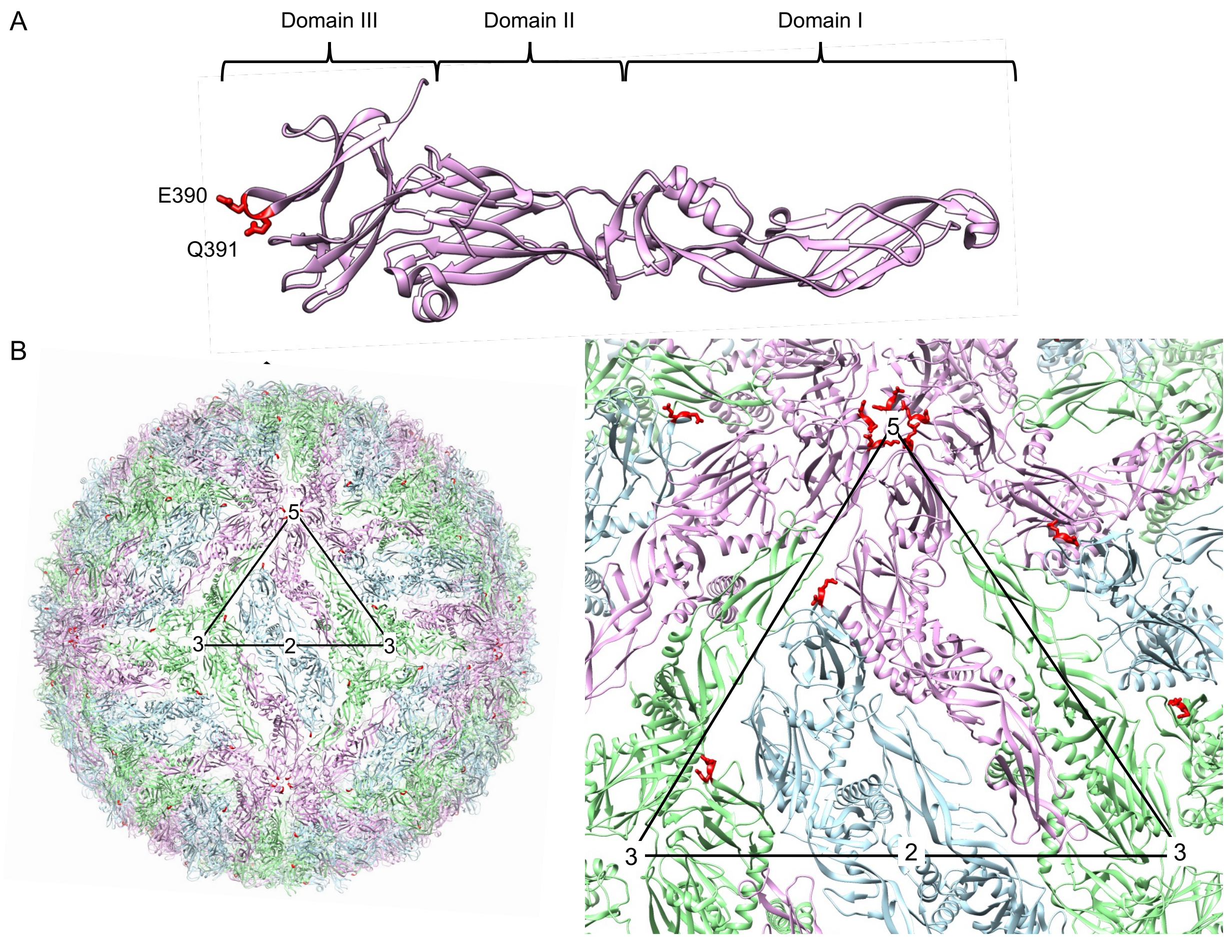

Fig S4. Envelope protein mutations identified from virus with SLA co-transfection. (A) Locations of non-synonymous mutations (E390G and Q391R, red) were mapped onto WNV envelope protein (PDB 7E4K). Both residues reside in the FG loop on the surface of domain III of envelope protein. **(B)** Locations of E390 and Q391 on the virion. E390 and Q391 are exposed on the surface of the icosahedral virion (PDB 7KVA) and are shown to be part of an epitope for neutralizing antibody binding (4). An asymmetric unit of the capsid is shown with the 5- and 3-fold regions labeled.

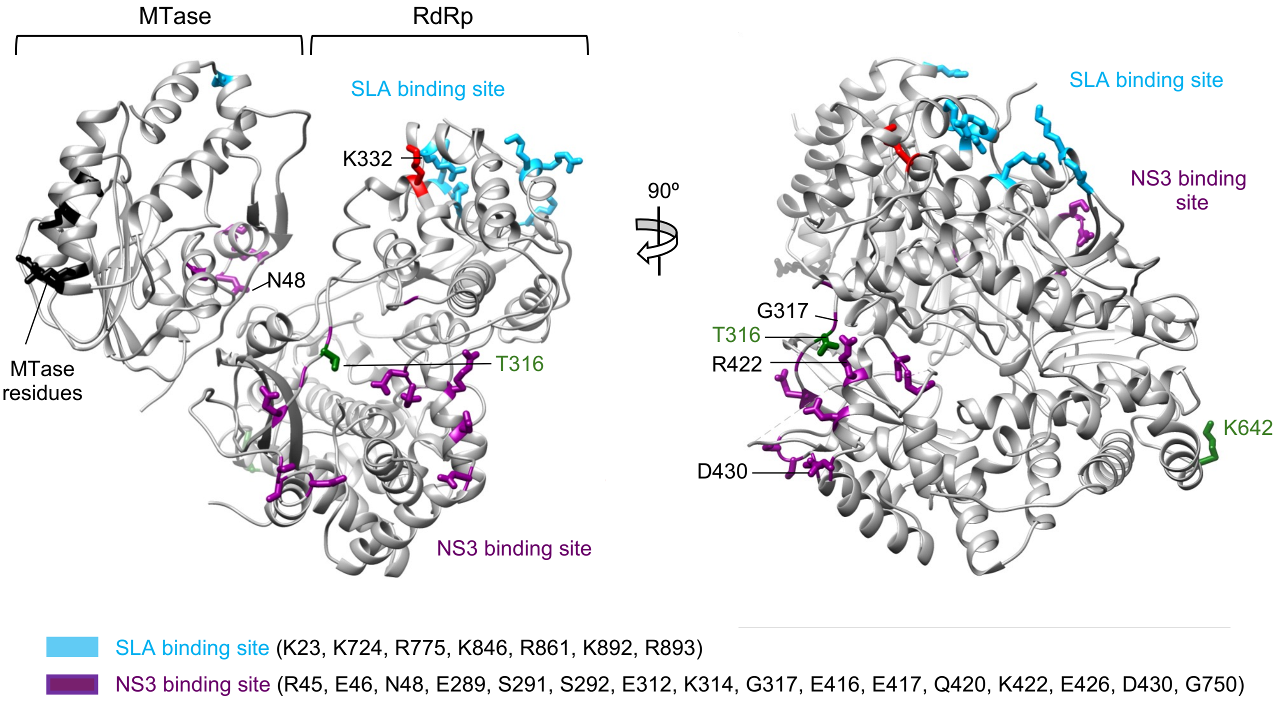

Fig S5. NS5 mutations identified from virus with SLA co-transfection. The full-length WNV NS5 was generated by overlaying the DENV promoter complex composed of NS5, NS3, and SLA (PDB 8GZQ) with individual WNV MTase (PDB 2OY0) and RdRp domain (PDB 2HFZ) in Chimera. Location of non-synonymous mutations (T316S and K642R, dark green) were mapped onto the WNV NY99 NS5 protein model. The previously identified SLA- and NS3-binding sites are shown in blue and magenta, respectively (1, 2). Among the NS3 binding residues, mutations of N48, G317, R422, and D430 (labeled) led to > 50% reduction in viral replication (2). Additionally, mutations of V191, K193, R200, and R201 in the MTase domain (colored in black) and K332 in the RdRp domain (red) abolish viral replication (2,3). T316 is located near the NS3-binding site but does not interact with NS3.

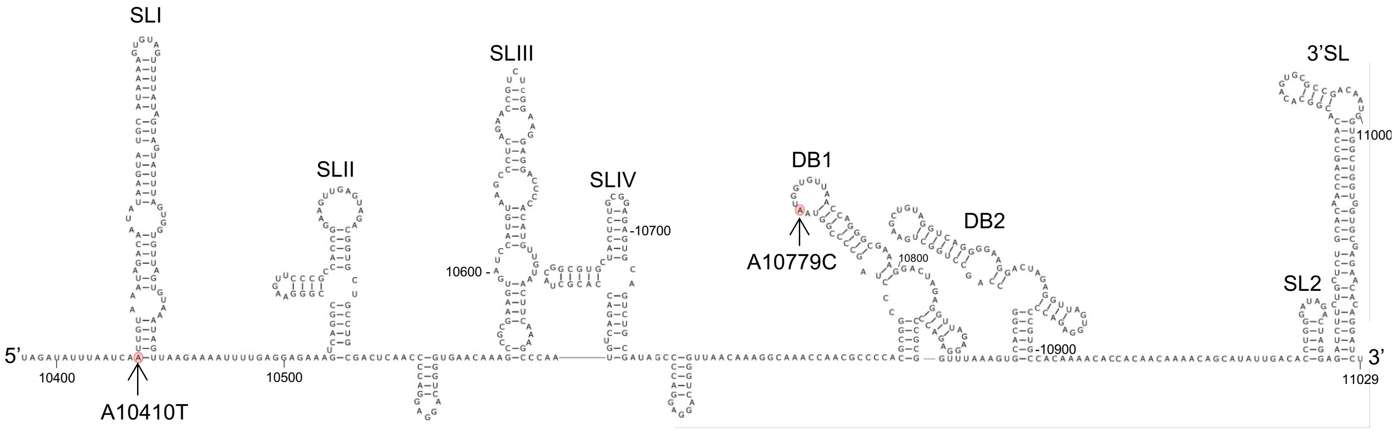

Fig S6. **3' UTR mutations identified from virus with SLA co-transfection.** The 3' UTR of the WNV genome contains four stem-loop structures (SLI-IV), two dumb-bells (DB1 and 2), and 3' terminal stem-loops (SL2 and 3'SL) (5,6). The locations of the A10410 and A10779 are mapped on the predicted secondary structures.

References

1. Osawa, T., Aoki, M., Ehara, H. and Sekine, S.I. (2023) Structures of dengue virus RNA replicase complexes. *Mol Cell*, **83**, 2781-2791 e2784.
2. Brand, C., Geiss, B.J. and Bisaillon, M. (2024) Deciphering the interaction surface between the West Nile virus NS3 and NS5 proteins. *Access Microbiol*, **6**.
3. Keating, J.A., Bhattacharya, D., Lim, P.Y., Falk, S., Weisblum, B., Bernard, K.A., Sharma, M., Kuhn, R.J. and Striker, R. (2013) West Nile virus methyltransferase domain interacts with protein kinase G. *Virology journal*, **10**, 242.
4. Kanai, R., Kar, K., Anthony, K., Gould, L.H., Ledizet, M., Fikrig, E., Marasco, W.A., Koski, R.A. and Modis, Y. (2006) Crystal structure of west nile virus envelope glycoprotein reveals viral surface epitopes. *J Virol*, **80**, 11000-11008.
5. Fernandez-Sanles, A., Rios-Marco, P., Romero-Lopez, C. and Berzal-Herranz, A. (2017) Functional Information Stored in the Conserved Structural RNA Domains of Flavivirus Genomes. *Front Microbiol*, **8**, 546.
6. Roby, J.A., Pijlman, G.P., Wilusz, J. and Khromykh, A.A. (2014) Noncoding subgenomic flavivirus RNA: multiple functions in West Nile virus pathogenesis and modulation of host responses. *Viruses*, **6**, 404-427.
